# A tale of two pandemics: The enduring partisan differences in actions, attitudes, and beliefs during the coronavirus pandemic

**DOI:** 10.1371/journal.pone.0287018

**Published:** 2023-10-25

**Authors:** Ying Fan, A. Yeşim Orhun, Dana Turjeman

**Affiliations:** 1 Department of Economics, University of Michigan, Ann Arbor, MI, Unted States of America; 2 Ross School of Business and School of Information, University of Michigan, Ann Arbor, MI, Unted States of America; 3 Arison School of Business, Reichman University, Herzliya, Israel; Xiamen University - Malaysia Campus: Xiamen University - Malaysia, MALAYSIA

## Abstract

Early in the new coronavirus disease (COVID-19) pandemic, scholars and journalists noted partisan differences in behaviors, attitudes, and beliefs. Based on location data from a large sample of smartphones, as well as 13,334 responses to a proprietary survey spanning 10 months from April 1, 2020 to February 15, 2021, we document that the partisan gap has persisted over time and that the lack of convergence occurs even among individuals who were at heightened risk of death. Our results point to the existence and persistence of the interaction of partisanship and information acquisition and highlight the need for mandates and targeted informational campaigns towards those with high health risks.

## 1 Introduction

Partisan differences have been documented in many policy domains. During the early months of the coronavirus pandemic, several studies also documented partisan gaps in self-reported precautionary behaviors and observed social mobility (e.g. [1–6]). At that time, partisan rhetoric was beginning to form about the seriousness of the pandemic, yet information about the effectiveness of different risk-mitigating strategies was scarce, ambiguous, and confusing. For example, the Centers for Disease Control (CDC) initially did not recommend masks, and many people were wiping down groceries. Scholars identifying partisan gaps in health-related behaviors and attitudes called for uniform public health messaging, in the hopes of eliminating the partisan gap through accurate and increased information dissemination.

Whether partisan gaps in attitudes, beliefs, and behaviors narrow with increased access to more accurate information remains an open question. On the one hand, we might expect gaps to decrease as more information becomes available. On the other hand, since partisan identity influences the selection and interpretation of information (e.g., [7, 8]), it is possible that initial partisan gaps may persist. In this paper, we study this question in the context of the coronavirus pandemic.

This context is ideal for studying the relationship between partisan gaps and information provision for three reasons. First, the pandemic is a high-stakes situation that poses direct personal risks, and the cost of being misinformed or guided by political ideology could be high to individuals and their loved ones. Second, accurate information about the virus and effective strategies to curb its spread became more widely available over the course of the pandemic. Third, there is heterogeneity in individuals’ incentives to acquire information. Being misinformed is more costly for older individuals and populations with pre-existing health risk factors.

Therefore, in this paper, we ask the following questions: Do partisan gaps in beliefs and behaviors regarding the pandemic narrow over time? Do partisan gaps narrow more for individuals who consume more information or for populations who face higher risks of complications and death?

To answer these questions, we document partisan gaps in behaviors, attitudes, and beliefs regarding the COVID-19 pandemic across a time span from the beginning of the pandemic to February 2021 and study their heterogeneity across individuals with different risk levels and information consumption habits. Our analyses primarily rely on an original and nationally representative survey conducted between April 1, 2020, and February 15, 2021, covering 13,334 respondents. Additionally, We analyze geo-location data from a large sample of smartphones provided by SafeGraph to examine differences in mobility due to both work and non-work trips.

We present three main findings. First, we show economically significant and persistent partisan gaps that emerge early in the pandemic and do not decrease over time. Using smartphone geolocation data, we document remarkably stable and large partisan gaps in mobility. Using our survey data, we also show partisan gaps in individuals’ self-reported behaviors (e.g., avoiding large gatherings or limiting seeing friends), attitudes towards economic activities (e.g., eating at a restaurant or going shopping), health worries (about themselves and those around them), and beliefs (about their own health risks and the future death toll of the pandemic in the U.S.) that are substantial and also persistent over time. Second, we document that partisan gaps are large and persistent even among individuals who are at a heightened risk of severe health outcomes (65 years or older, or have pre-existing health conditions that put them in the high-risk category). For this high-risk population, accurate information is highly valuable. However, we find that although for some measures the partisan gap among the high-risk population is somewhat smaller than that among the low-risk population during some periods, the gaps among the high-risk population across all measures (on protective behaviors, comfort with economic activities, and beliefs) are still substantial and do not decrease over time. Third, individuals who are exposed to news outlets within a narrower political spectrum and those who choose not to pay much attention to information about the pandemic are responsible for the documented partisan gaps. The partisan gaps are largely nonexistent among individuals who choose to follow news outlets that span the political spectrum and are also greatly reduced among individuals who report paying more attention to information about the pandemic from a variety of formal and informal sources.

Together, our results suggest that making more information available is important, but not sufficient, to narrow partisan gaps. On the one hand, partisan gaps are absent among individuals who consume information about the pandemic, which underscores the importance of information provision. On the other hand, the availability of accurate information does not ensure that individuals utilize it, as indicated by enduring partisan gaps in beliefs, attitudes, and behaviors over the long run, even among at-risk populations. Along with the well-known political divide in news consumption and the persistent slant in news supply (e.g., [9, 10]), these results further highlight the difficulty of relying on free-market news and individual rationality to eradicate partisan gaps over time and advocate for targeted measures to increase information consumption, especially among individuals with high health risks. We also find that partisan gaps are reduced for certain behaviors during periods that mandate these behaviors (e.g., social distancing, mask-wearing). These results underscore the usefulness of universal mandates in protecting vulnerable populations.

This paper contributes to two strands of literature. First, this paper adds to a large literature on partisan gaps or partisan bias in economics. Examples include [11] on the effect of gubernatorial partisanship on local policies and economic outcomes [12], on political bias in newspaper coverage of economic issues [13], on the effect of partisan ideology on public healthcare expenditure [14], on partisan bias in the investment and the performance of state pension funds, and [15] on partisan bias in economic expectations and household spending. Among this literature, it is most related to the recent set of papers that document partisan gaps in individual behaviors and beliefs during the early phase of the COVID-19 pandemic. Examples include [1–6, 16, 17]. Our paper contributes to this literature through a combination of observational data analysis and survey work that spans a wider set of questions regarding attitudes, beliefs, and behaviors and a much longer period of time. Where there is overlap, our results are broadly consistent with earlier work regarding the early phase of the pandemic. We further contribute to this literature by (1) documenting the persistence of partisan gaps in individuals’ actions, attitudes, and beliefs, and (2) exploring the heterogeneity in partisan gaps.

Second, this paper contributes to a nascent literature providing field evidence of persistent belief biases. Although experimental studies have shown that initial beliefs influence individuals’ learning process, it is unclear whether the systematic belief biases discussed in this literature persist in the long run and exist in high-stakes contexts—a question that, by its nature, needs to be examined in the field. Several field studies have shown evidence of biased beliefs in contexts where individuals presumably observe signals that should challenge their beliefs. For example [18–20], document persistent overconfidence among chess players, truckers, and managers regarding their ability or performance. We contribute to this literature by providing field evidence on the extent to which disparate beliefs persist over a long period of time in an information-rich environment where mistakes can have life-or-death consequences. We show that even the most vulnerable members of the population sustain belief and behavior gaps along partisan lines, despite facing very high personal stakes.

## 2 Materials and methods

We use two sources of data. First, we analyze geolocation data reflecting mobility choices in the U.S. Second, and centrally, we analyze an original and nationally representative survey of behaviors, attitudes, and beliefs regarding the pandemic. We describe these data sources and our methods sequentially.

### 2.1 Mobility data and analysis

#### Data

SafeGraph is a data company that aggregates anonymized location data from numerous smartphone applications to provide insights about physical places (www.safegraph.com). Using geo-location data obtained from the activities of more than 35 million smartphones, it provides an aggregated dataset (Social Distancing Metrics) to researchers. For each census block on a given day, the Social Distancing Metrics data report the total number of devices, the number of devices exhibiting full-time work behavior (spending more than 6 hours between 8 a.m. and 6 p.m. in one location outside the home), devices exhibiting part-time work behavior (spending more than 3 but less than 6 hours between 8 a.m. and 6 p.m. in one location outside the home), and devices that stay home all day. SafeGraph determines the home of a device by its common nighttime location over a 6-week period. We aggregate this data to the county-day level and merge it with county data on demographics, election results from the 2016 presidential election, daily local COVID-19 cases and deaths, and daily minimum and maximum temperature, precipitation, and wind speed. We exclude Alaska and territories of the U.S. Moreover, following SafeGraph warnings, we drop two dates (2/25/2020 and 1/27/2021) with anomalous data. To enhance privacy, SafeGraph excludes a census block group if it has fewer than five devices observed in a month. We obtain county-level demographic information from [21], 2016 presidential election results from [22], COVID-19 case and death numbers from [23], and weather data on temperature, precipitation, and wind speed from gridMET as maintained by [24].

#### Methods

We examine how the shares of devices that left home differ across counties of varying political slant between February 2, 2020, and February 7, 2021. Our main specification is:

$$\begin{array}{c} s_{ct}=P_{c}\beta_{w(t)}+D_{ct}\gamma_{w(t)}+\zeta_{c}+\eta_{s(c)t}+\varepsilon_{ct}, \end{array}$$ (1)

where *s*_*ct*_ is the share of devices in county *c* on day *t* exhibiting a particular behavior. Specifically, we calculate the shares of devices that left home for any reason, for full-time work, for part-time work, or for non-work reasons using the total number of devices in the sample as the denominator and the number of devices exhibiting each of these behaviors as the numerators. SafeGraph reports a sampling bias in favor of detecting devices that are moving (see [25]). S1 Appendix shows the robustness of our conclusions to several different adjustment approaches.

The independent variable of interest, *P*_*c*_, is the percent voting for Clinton in the 2016 election. We use this vote share as a proxy to capture a county’s political slant. The coefficients of interest, *β*_*w*(*t*)_, are allowed to vary by week *w*(*t*). We also let control variables *D*_*ct*_ to vary weekly in their influence. They include demographic and socioeconomic controls, daily weather controls, and variables to capture differences in COVID-19 risks across counties. Demographic control variables are percent living under the poverty line, the population share of women, the population share of individuals living in poverty, population shares of whites, blacks, Asians and other non-whites, percent of adults with a bachelor’s degree, percent of adults without a high-school degree, the unemployment rate, and the employed share of the county population. Weather controls include daily minimum and maximum temperature, precipitation, and wind speed. Pandemic risk proxies include the logarithm of one plus the number of COVID-19 cases, the logarithm of one plus the number of COVID-19 deaths, the logarithm of population density, population share over the age of 65, and a binary variable indicating whether the county is in a metropolitan area. We include county fixed effects (*ζ*_*c*_) to control for cross-sectional differences and state-date fixed effects (*η*_*s*(*c*)*t*_) for state-wide changes in social-distancing policies. Because each observation is a share in a county, we weigh observations by the total number of devices in each county. We cluster errors at the county level.

### 2.2 Survey data and analyses

In addition to studying partisan gaps in social mobility using the SafeGraph data, we also conduct a survey and use the survey data to examine partisan differences in actions, attitudes, and beliefs that are not possible to observe directly.

#### Data

The survey data comprises 13,334 responses to a nationally representative (in terms of age, ethnicity, gender, income, and geographic regions) survey of the U.S. adult population. We used https://luc.id/theorem/ to target a nationally representative sample. We obtained respondent demographics (age, race/ethnicity, gender, educational level, annual household income, presence of children at home, and political affiliation) from Lucid. Table S2.1 in S2 Appendix confirms that our sample tracks well with the U.S. national benchmarks in age, race/ethnicity, gender, and regions of the U.S., albeit slightly under-sampling whites. The survey was fielded across 5 time periods between April 1, 2020, and February 15, 2021: early April 2020, late April 2020, June 2020, August 2020, and February 2021. We map zip codes to counties and merge them with the external datasets to obtain county-level population density, COVID-19 infection, and death counts. The University of Michigan Institutional Review Board (IRB) reviewed the surveys and determined that they are exempt from ongoing review (HUM00148129, HUM00180582). The IRB has also approved the merge between survey responses and county-level data (HUM00180640).

The survey asked respondents about their adoption of protective behaviors, comfort with engaging in certain activities after stay-at-home mandates were lifted, worries regarding health and economic well-being, and beliefs regarding the impact of the virus. The survey also elicited pre-existing health risks, the role of constraints as a driver of personal choices during the pandemic, news consumption habits, and the amount of attention respondents paid to different sources of information regarding the pandemic. S3 Appendix presents the survey instrument.

#### Methods

We first study partisan gaps across time in protective behaviors, comfort with different activities, health worries, and beliefs. We then study how partisan gaps in these measures vary with the individuals’ health risks, consumption of bipartisan news, and level of attention to information about the pandemic.

To document partisan gaps over time, we use the following regression equation:

$$\begin{array}{c} Y_{i\tau }=P_{i}\alpha_{\tau }+X_{i\tau }\lambda_{\tau }+\mu_{s(i)\tau }+\varepsilon_{i\tau }, \end{array}$$ (2)

where *Y*_*iτ*_ is an outcome variable of interest for person *i* at time *τ*. There are four groups of outcome variables, related to, respectively, protective behaviors, comfort with different activities, worries, and beliefs. First, to elicit protective behaviors, the survey asked “Which of the following changes have you personally made to protect yourself from the coronavirus infection?” for a set of behaviors including: (1) do not meet friends or extended family; (2) avoid large gatherings and public transportation; (3) wash hands more often; and (4) wear a mask when out and about. The first two are social distancing actions that may contribute to the mobility differences we also examine with observational data, and the latter two are health precautions individuals were encouraged to take.

Second, to elicit comfort with activities, from June 2020 on, as most of the stay-at-home orders were lifted across the nation, the survey asked respondents whether or not they “feel comfortable in engaging in” the following activities: (1) eat in a restaurant with indoor seating; (2) eat in a restaurant with outdoor seating; (3) be part of a gathering with more than 10 people; (4) go to a coffee shop; (5) go to a bar; (6) go to a gym; (7) go grocery shopping; and (8) go shopping for non-food items. For both protective behaviors and comfort with activities, the dependent variables are coded as indicators (0/1) on each of the items.

Third, the survey asked how worried respondents felt about their own health, and the health of their partner, kids, extended family, members of their community, and the whole U.S. (on a scale of 1 to 5, 1 being not worried at all, 5 being extremely worried). It also asked how worried they feel about the economic well-being of the same groups of people (using the same scale).

Fourth, the survey elicited two types of beliefs: (1) Individuals’ predictions on their own health risks, i.e., the chance of becoming infected in the next three months and the chance of having no/mild or serious symptoms should they get infected. (2) Their predictions of the number of U.S. deaths by a certain target date assuming the state policies remain the same. The target date was July 1, 2020, in the April waves, September 1, 2020, in the June waves, December 1, 2020, in the August wave, and May 1, 2021, in the February wave. For worries and beliefs, we use standardized (z-score) measures as dependent variables.

As for the independent variables, *P*_*i*_ is a vector of indicator variables representing person *i*’s political affiliation (Democrat, Republican, or Independent). We allow the impact of partisanship, captured by *α*_*τ*_, to vary over time. We determine respondents’ political affiliation as Democrats, Republicans, and Independents based on their responses to the political affiliation question asked by Lucid. We indicate an individual as a Democrat (Republican) if they choose one of the following responses to the political affiliation question: “Strong Democrat (Republican),” “Not very strong Democrat (Republican),” “Independent Democrat (Republican)” or “Other—leaning Democrat (Republican).” We indicate an individual as Independent if they choose one of the following responses to the political affiliation question: “Independent—neither” or “Other—neither.” Alternative specifications of this variable do not substantively change our results.

The control variables *X*_*iτ*_ include individual-specific demographic controls (categorical variables for the respondent’s gender, race, age, educational level, annual household income, and presence of children) and an indicator for whether the respondent has any of the chronic conditions listed by the CDC as a high-risk factor. Control variables *X*_*iτ*_ also include geography-specific covariates that proxy for local pandemic severity over time (natural logarithm of one plus the number of cumulative COVID-19 cases in the respondent’s county at the time of the survey, natural logarithm of one plus the number of cumulative deaths in the respondent’s county at the time of the survey, natural logarithm of the population density of the respondent’s zip code). Summary statistics of control variables are in Table S2.2 of the S2 Appendix. We include state-time fixed effects, *μ*_*s*(*i*)*τ*_, to capture systematic differences across states at the time.

In addition to studying the partisan gaps over time using Eq (2), we also examine whether these gaps and their persistence are heterogeneous across individuals with different health risks, bipartisan news consumption, and attention to information about the pandemic using Eq (3) below:

$$\begin{array}{c} Y_{i\tau }=\alpha_{\tau }P_{i}+\beta_{\tau }Z_{i}+\gamma_{\tau }\left(P_{i}\cdot Z_{i}\right)+X_{i\tau }\lambda_{\tau }+\mu_{s(i)\tau }+\varepsilon_{i\tau }, \end{array}$$ (3)

where *Y*_*iτ*_ (the outcome variable of interest), *P*_*i*_ (the political affiliation dummy variables), the control variables *X*_*iτ*_ and *μ*_*s*(*i*)*τ*_ (the state-period fixed effects) are the same as those in Eq (2). *Z*_*i*_ is a binary variable capturing individual heterogeneity of interest. In this specification, the coefficient *α*_*τ*_ gives the partisan gaps among individuals for whom *Z*_*i*_ = 0, and the sum *α*_*τ*_ + *γ*_*τ*_ measures that for individuals for whom *Z*_*i*_ = 1.

We examine three dimensions of heterogeneity. First, we study differences between individuals with high or low health risks. We consider an individual to be at a higher health risk (*Z*_*i*_ = 1) if they are either 65 years or older or have at least one pre-existing health condition that qualifies them as at-risk by the CDC, and at lower risk otherwise (*Z*_*i*_ = 0). The survey asked respondents to indicate any conditions they have from a list of diseases obtained from the CDC website on March 30, 2020: Moderate to severe asthma; COPD or other chronic lung diseases; Serious heart conditions; Diabetes; Conditions that can cause a person to be immuno-compromised including cancer treatment, smoking, bone marrow or organ transplantation, immune deficiencies, poorly controlled HIV or AIDS, and prolonged use of corticosteroids and other immune weakening medications; Severe obesity (BMI of 40 or higher); Chronic kidney disease and currently undergoing dialysis; Liver disease. 57% of our respondents are considered high-risk. Second, we examine differences between individuals who consume news across vs. along partisan lines. The survey asked individuals to indicate the top news outlets they regularly consume. We define an individual as consuming bipartisan news (*Z*_*i*_ = 1) if the top news sources they regularly report consuming span the liberal-conservative divide, or as consuming news along partisan lines (*Z*_*i*_ = 0) otherwise. While there are partisan differences in choices of news outlets, a considerable fraction of people consume news across the partisan line: 46% of Democrats indicate Fox News as a source they regularly watch, and 42% of Republicans consume a more liberal news outlet in addition to Fox News. We consider CNN, NBC/MSNBC, NPR, Huff Post, The New York Times and Washington Post as liberal news outlets, but the results are robust to narrower classifications. Third, we examine differences between individuals who pay more vs. less attention to information about the pandemic. The survey asked participants to indicate the amount of attention they pay to various information sources (on a 5-point scale ranging from 1 “not at all” to 5 “very much so.”) spanning from scientists/researchers to friends and family members regarding the pandemic specifically. A respondent is said to pay more attention to information about the pandemic (*Z*_*i*_ = 1) if their average attention score across different information sources is above 3.5 on a 5-point scale, and as paying less attention (*Z*_*i*_ = 0) otherwise. Again, while there are partisan differences in information attention, there are within-Democrats and within-Republican variations in the amount of overall attention they pay to others in terms of the pandemic, calculated as the average attention score across all items. Summary statistics of news consumption and attention to different sources broken down by political affiliation can be found in Table S2.3 in S2 Appendix.

## 3 Results

### 3.1 Partisan gaps in mobility

**Fig 1:**
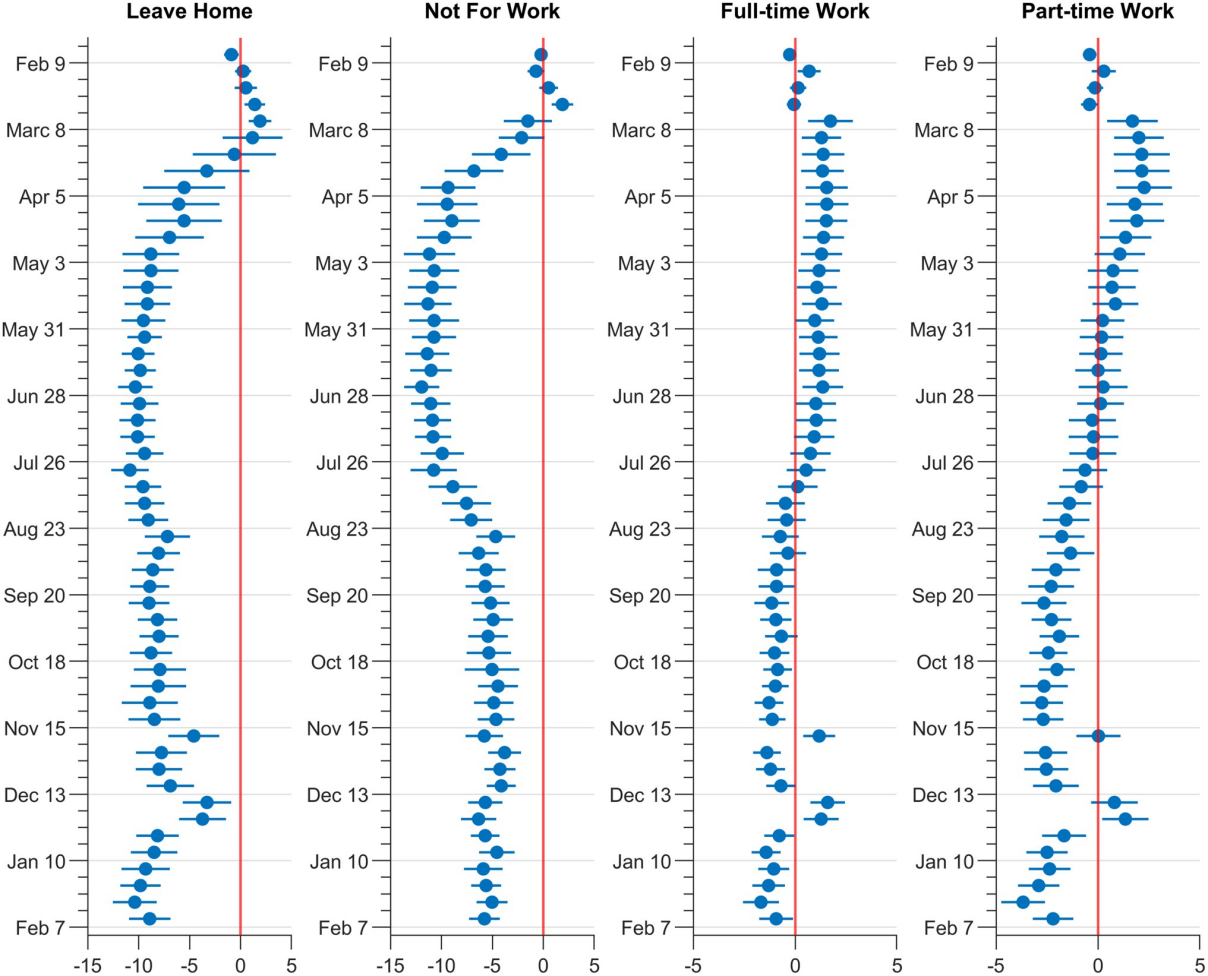
Partisan gaps in the shares of devices engaging in different types of activity. Notes: This figure plots the estimated coefficient *β*_*w*(*t*)_ in Eq (1) and the corresponding 95% confidence interval where the dependent variable is indicated at the top of each panel. The week of February 1, 2020, is taken as the baseline *t* = 0. The y-axis indicates the beginning date of the week for which the coefficients are reported. Observations are weighted by the number of candidate devices in the county, and standard errors are clustered at the county level.

Fig 1 presents the estimated coefficient *β*_*w*(*t*)_ from regression Eq (1). Assuming that differences across counties in the voting shares of the 2016 election are highly correlated with differences across counties in political affiliation during the pandemic, *β*_*w*(*t*)_ reflects the partisan gap in county mobility in week *t*. Each panel of the figure corresponds to a share of devices exhibiting a certain behavior: leaving home for any reason, for non-work reasons, for full-time work, or for part-time work. The coefficients are estimated relative to the first week in our sample—the week of February 2, 2020. For historical context, recall that the first confirmed case in the U.S. was reported on January 21, 2020, travel from China to the U.S. was banned on February 2, travel restrictions were extended to Iran on February 28 and to European nations on March 12, 2020.

From the first panel of Fig 1, we can see that while differences in mobility across counties with varying degrees of political slant were nonexistent during February 2020, these differences emerge quickly in early March and remained stable until the end of our sample. The estimated *β*_*w*(*t*)_ hovers around -9 throughout the study period. To put this estimate into context, we note that the difference between the 95th and 5th percentiles of Democratic vote share across counties is 0.5. Therefore, an estimated value of -9 for the coefficient *β*_*w*(*t*)_ implies that going from a county with the 5th to the 95th percentile in Democratic vote share is associated with a decrease of 4.5 percentage points in the share of devices staying at home. Such a partisan gap is sizable compared to the overall change in mobility. During the first week of the sample, 74.6% of devices left home, while in July and August of 2020, 67.8% of devices left home on average. Therefore, a voting share gap of 4.5 percentage points in the 2016 election is equivalent to 66% of the mobility reduction from the first week of the sample to the summer of 2020.

Looking into various reasons why people leave home, we can see that the partisan gap in discretionary (non-work) mobility (the second panel of Fig 1) dominates partisan gaps in work-related mobility (the third and fourth panels of Fig 1) and the former drives the persistent partisan gap in the overall mobility. In particular, the partisan gap in social mobility for non-work purposes starts in early March, stabilizes in April, and remains at that level throughout the summer of 2020. Afterward, when the school year starts and when many workers return to their workplaces as economic activities resume across the nation, the partisan gaps in non-work mobility become smaller but never disappear. Turning to work-related mobility, we find that partisan gaps are much smaller, but also display patterns consistent with Democrats reducing mobility more when they can. Specifically, we find that people in counties with a higher Democratic vote share are more likely to leave home for work when the economy is closed (before the end of the summer) and during holidays (Thanksgiving, Christmas), but less likely to do so as a larger part of the population returns to their workplaces (after the summer). While the former pattern is consistent with the possibility of a higher representation of Democrats in front-line professions that require working outside the home (e.g. healthcare, retail, and grocery stores), the contrast between the former and the latter patterns means that barring constraints imposed by workplaces and professions, Democrats seem more likely to reduce mobility for work purposes. In S1 Appendix, we confirm a positive correlation between the share of most front-line occupations and Democratic vote share in a county, and report results from a specification that includes occupation share controls. Results are presented in Fig S1.1 in S1 Appendix. As expected, the partisan gaps in mobility associated with work trips during the early phase of the pandemic are less pronounced with these controls.

### 3.2 Partisan gaps in individual actions, attitudes, and beliefs

#### Persistence in partisan gaps

**Fig 2:**
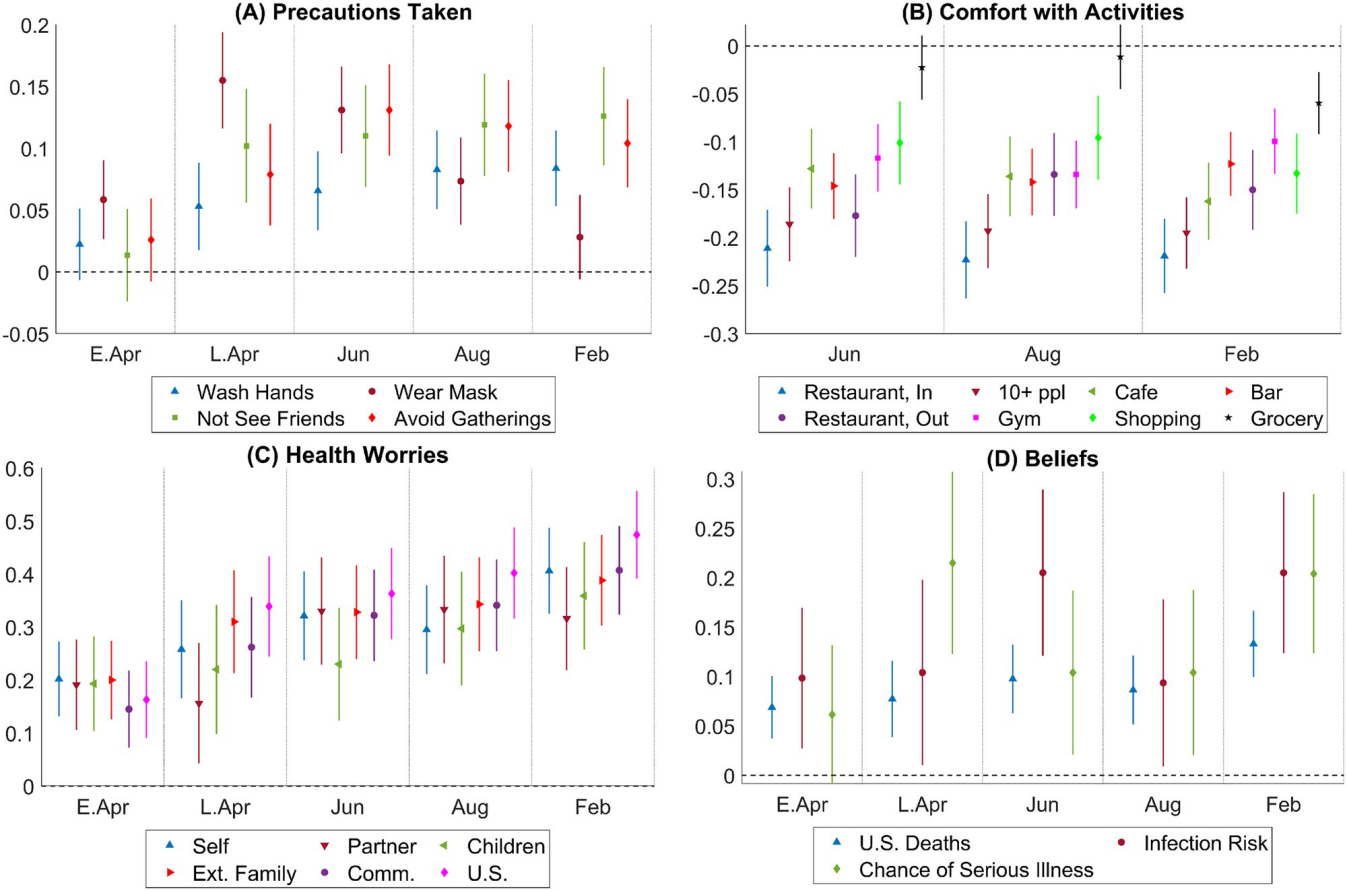
Partisan gaps in actions, attitudes, worries, beliefs. Notes: This figure plots the estimated Democrat—Republican partisan gaps obtained from the estimates of *α*_*τ*_ in Eq (2) and the corresponding 95% confidence intervals. The x-axis indicates the period *τ*. In Panel A, a positive estimate means that, *ceteris paribus*, Democratic respondents are more likely than Republican respondents to have taken an action indicated in the legend. The actions studied are “Wash Hands”–wash hands more often; “Wear Mask”– wear a mask when out and about; “Not See Friends”– do not meet any friends or extended family; “Avoid Gatherings”– avoid public transportation and large gatherings. In Panel B, a positive estimate means that, *ceteris paribus*, Democratic respondents are more likely than Republican respondents to feel comfortable with an activity indicated in the legend. Activities studied are “Restaurant, In”– eat in a restaurant with indoor seating; “Restaurant, Out”– eat in a restaurant with outdoor seating; “10 + ppl”– be part of a gathering with more than 10 people; “Cafe”– go to a coffee shop; “Bar”– go to a bar; “Gym”– go to a gym; “Shopping”– go shopping for non-grocery items; “Grocery”– go grocery shopping. In Panel C, a positive estimate means that, *ceteris paribus*, Democratic respondents worry more about the health well-being of the group of people indicated in the legend. The groups of people are “Self”– respondent herself; “Partner”– respondent’s partner; “Children”– respondent’s kids; “Ext. Family”– respondent’s extended family; “Comm.”– members of the respondent’s community; “U.S.”– all people in the U.S. In Panel D, a positive estimate means that, *ceteris paribus*, Democratic respondents predict a larger number on the outcomes indicated in the legend. Outcomes over which expectations are elicited are “U.S. Deaths”– total number of deaths in the U.S. by a target date; “Chance of Infection”– chances that the respondent will get infected with the coronavirus in the next three months; “Chance of Serious Illness”– chances that the respondent will have serious symptoms should she get infected.

Fig 2 plots the Democratic—Republican partisan gaps obtained from the estimates of *α*_*τ*_’s in regression Eq (2). Differences between Independents and Republicans are more muted overall, and are reported in Fig S2.2 in S2 Appendix.

Panel A depicts the estimated differences between Democrats and Republicans in the probability of taking protective actions. From this panel, we can see that although partisan gaps in social distancing and washing hands are negligible in the early days of the pandemic, they quickly grow to a substantial level by the end of April 2020 and remain high even by February 2021. The absence of partisan gaps in social distancing early in the pandemic corresponds with stay-at-home mandates many states had in place. As these restrictions are lifted, partisan gaps emerge. Regarding mask-wearing, a small partisan gap is present early in the pandemic, grows larger during the summer of 2020, and is diminished greatly by February 2021 when most businesses and local governments mandate masks indoors.

Panel B of Fig 2 presents partisan differences in comfort with various economic activities. Democrats are less likely to feel comfortable with engaging in economic activities overall. Except in the case of grocery shopping, the partisan gap is consistently between 10% and 20% across these activities, and stably so over time. These partisan gaps are substantial in magnitude. For example, over time, the overall share of respondents reporting feeling comfortable with dining indoors increased from 29% in June 2020 to above 42% in February 2021 (see Fig S2.1 in S2 Appendix). A partisan gap of 20% is, therefore, equivalent to about half of the overall change in comfort with indoor dining over time.

As Panel C of Fig 2 shows, from the beginning of the pandemic to the last period of our survey, Democrats report being more worried than Republicans about their own health that of their partner’s, local community’s, and the whole nation’s. The partisan gap in health worries varies between 20–40% of a standard deviation, and increases over time. Fig S2.3 in S2 Appendix depicts the estimated partisan gaps for economic worries, which are statistically significant and in the same direction, but more muted in magnitude.

Finally, Panel D of Fig 2 presents partisan gaps in predictions regarding own or systemic health risks. From the figure, we can see that while the magnitude of the gaps varies over time, the gaps are almost always present: Democrats are consistently more pessimistic than Republicans. For example, despite being more likely to take precautionary actions, Democrats’ prediction of their chance of becoming infected in the next three months from the time of the survey is, on average, 0.1 to 0.2 standard deviations higher than that of Republicans. Their predictions about their chance of getting serious symptoms conditional on getting infected are also 0.1 to 0.2 standard deviations higher. In terms of systemic risks, Democrats on average predict more deaths due to COVID-19 than Republicans by around 0.1 standard deviations.

Overall, the above results show that throughout the 10-month period, Democrats were consistently more worried about the health impact of the pandemic, more cautious about engaging in economic activities, and congruently, more likely to take social distancing and preventive actions. One might be concerned that partisan differences in health behaviors existed prior to the pandemic. Note that questions regarding social distancing, seeing friends and comfort with different activities are specific to the pandemic itself. Therefore, this concern would center around potential pre-existing partisan gaps in hand-washing or in health worries. According to our results, partisan gaps in hand-washing and health worries were small in early April but increased over time, implying that pre-existing partisan gaps, if there were any, cannot account for the results entirely.

#### Heterogeneity in partisan gaps

**Fig 3:**
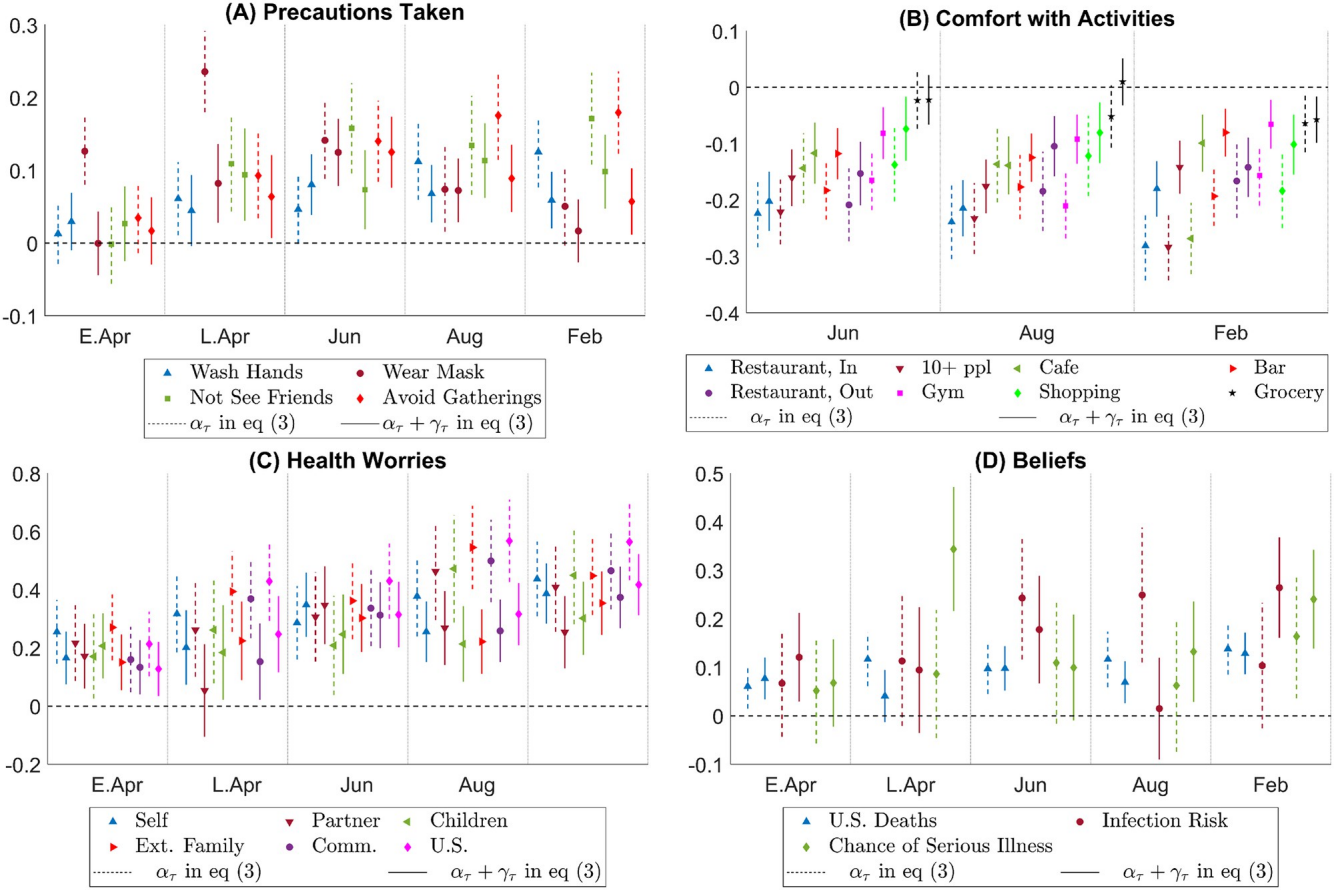
Partisan gap heterogeneity across high-risk and low-risk individuals. Notes: This figure plots the estimated Democrat—Republican partisan gaps for low-risk and high-risk respondents. In each panel, a dashed line and the marker on it give the confidence interval and the estimate for *α*_*τ*_ in Eq (3), which is the partisan gap among low-risk respondents, and a solid line and the marker on it give the confidence interval and the estimate for *α*_*τ*_ + *γ*_*τ*_, which is the partisan gap among high-risk respondents. The x-axis indicates the period *τ*. The legends are the same as those in Fig 2.

Fig 3 plots the estimated Democrat—Republican partisan gap for low-risk individuals and high-risk individuals separately, i.e., the estimated *α*_*τ*_ and *α*_*τ*_ + *γ*_*τ*_ in Eq (3) where *Z*_*i*_ is 1 for individuals with high risks and 0 otherwise. The estimates reveal two patterns: first, across all variables of interests (precautionary actions, attitude towards economic activities, health worries, and beliefs), the partisan gap among high-risk individuals is often statistically the same as (and sometimes smaller than) the partisan gap among low-risk individuals. Second, partisan gaps among high-risk individuals are substantial and stable over time. For example, high-risk Democrats are more likely to avoid seeing friends than high-risk Republicans by 10 percentage points (Panel A) and less likely to feel comfortable with a gathering with more than ten people by more than 10 percentage points (Panel B) throughout the sample. They are also more worried (Panel C) and hold more pessimistic beliefs (Panel D) than their Republican counterparts. These results are alarming as they indicate that as information about the disease becomes more accurate and widespread, partisan gaps remained strong even for the most vulnerable individuals.

**Fig 4:**
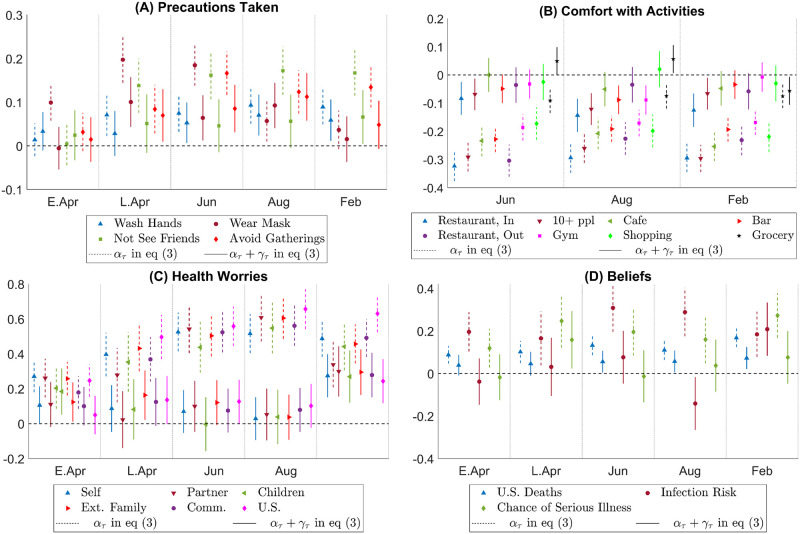
Partisan gap heterogeneity across those who consume news across the political line vs. those who do not. Notes: This figure plots the estimated Democrat—Republican partisan gaps for respondents who consume news across the political line and for those who do not. In each panel, a dashed line and the marker on it give the confidence interval and the estimate for *α*_*τ*_ in Eq (3), which is the partisan gap among consumers of narrow news, and a solid line and the marker on it give the confidence interval and the estimate for *α*_*τ*_ + *γ*_*τ*_, which is the partisan gap among consumers of news across the political line. The x-axis indicates the period *τ*. The legends are the same as those in Fig 2.

Fig 4 plots the estimated Democrat—Republican partisan gap for individuals who consume news across the partisan line versus those who consume a politically narrow scope of news, i.e, the estimated *α*_*τ*_ and *α*_*τ*_ + *γ*_*τ*_ in Eq (3) where *Z*_*i*_ is 1 for individuals consuming news across the partisan line and 0 otherwise. We find that throughout the period we study, partisan gaps in precautions taken, comfort with economic activities, health worries, and beliefs among individuals who choose to consume news across the partisan line are small in magnitude and oftentimes insignificant. In contrast, among individuals who do not consume news across the partisan line, while partisan gaps in actions and health worries quickly increase and become the main driver of partisan gaps. Moreover, partisan gaps in comfort with economic activities and beliefs are also large and significant in this group.

**Fig 5:**
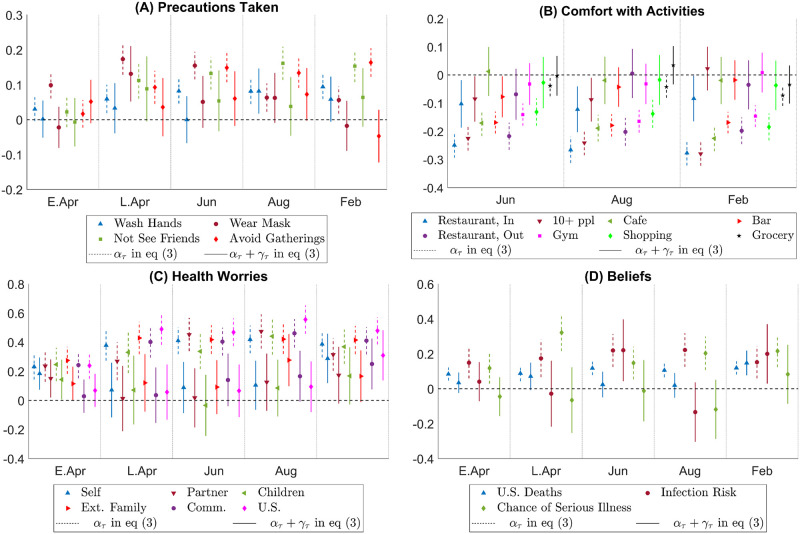
Partisan gap heterogeneity across those who pay attention to other information sources vs. those who do not. Notes: This figure plots the estimated Democrat—Republican partisan gaps for respondents whose average attention score on a 1–5 scale across other information sources (Friends, Family, Scientists, Pastor, Facebook or Twitter, CDC, Governor, President) is greater than 3.5, and those whose average attention score is 3.5 or lower. In each panel, a dashed line and the marker on it give the confidence interval and the estimate for *α*_*τ*_ in Eq (3), which is the partisan gap among those who do not pay a lot of attention to other information sources, and a solid line and the marker on it give the confidence interval and the estimate for *α*_*τ*_ + *γ*_*τ*_, which is the partisan gap among those who pay high attention overall. The x-axis indicates the period *τ*. The legends are the same as those in Fig 2.

Fig 5 depicts the estimated Democrat—Republican partisan gap for individuals who pay attention to information about the pandemic versus those who do not. The results show that among individuals who pay a high level of attention to information from others regarding the pandemic (average attention score greater than 3.5 on a scale of 1 to 5), the partisan gaps in precautions taken, comfort with economic activities, health worries and beliefs are economically small and oftentimes insignificant. In contrast, individuals who report not paying much attention overall to others as a source of information about the pandemic show substantial partisan gaps in all metrics.

## 4 Conclusion

### 4.1 Discussion and implications

Analyzing mobility data, we find that partisan gaps in overall mobility are nonexistent before the pandemic, arise quickly at the beginning of the pandemic, and are economically meaningful and persistent over time. These gaps are mainly driven by non-work-related mobility, which tends to be more discretionary. Using survey data, we examine the individual behaviors driving differences in discretionary mobility (e.g., social distancing, not seeing friends) and find partisan gaps in these behaviors. In addition, we also find partisan gaps in other protective behaviors as well as individuals’ attitudes and beliefs. These gaps remain persistent over a 10-month period.

What might explain such persistent partisan gaps? We first note that there are significant and stable associations between individuals’ risk beliefs and their actions and attitudes. For example, we find a positive association between disease severity beliefs and whether or not a person engages in a given protective behavior, and a negative correlation between disease severity beliefs and feeling comfortable engaging in economic activity (Fig S2.4 in S2 Appendix. In S2 Appendix, we also show positive and sustained correlations with infection risk beliefs and death toll beliefs and offer a more detailed discussion.) A one-standard deviation increase in the perceived chance of serious illness is associated with a 2.9%—8.5% increase in self-reported refraining from seeing friends across the 10-month span, and a 5%-7.6% decrease in whether the individual is comfortable with dining indoors. Moreover, the associations between risk perceptions and protective behaviors and comfort with economic activities are stable over time. An exception is the correlation between beliefs and wearing masks. In the early periods of the pandemic, the correlation is substantial, but as mask mandates are instituted, this behavior-belief correlation disappears. While we refrain from a causal interpretation, we note that a sustained association between behaviors and beliefs over a 10-month span is suggestive of the possibility that the health risk assessment differences across the partisan line are at least partially responsible for the behavioral differences.

What leads to the differences in beliefs and attitudes (and hence behaviors)? Our results on the heterogeneity in partisan gaps across consumers with different news consumption habits and information attention levels indicate that partisan gaps are absent among individuals who are exposed to news outlets of a wide political spectrum and individuals who pay attention to information from others regarding the pandemic. For this group, while a paucity of information early on may have allowed for initial heterogeneity in health worries, partisan gaps were eliminated quickly over time. This finding points to the importance of information in closing partisan gaps.

Unfortunately, at the same time, we find that there is no significant difference between the partisan gaps among high-risk individuals and those among low-risk individuals in terms of both the level (how big the gaps are) and the change (how the gaps change over time). High-risk individuals should have greater incentives to acquire information instead of being guided by political ideology. Models of rational information acquisition and updating would predict smaller gaps and faster convergence for this population. However, as information becomes more widespread, the partisan gap remains stable, even among this population. This implies that the increase in information supply did not guarantee higher consumption of information, and greater incentives for being informed did not narrow partisan gaps either.

Taken together, the importance of information, the lack of difference in partisan gaps among high-risk vs. low-risk individuals, and the persistence in partisan gaps suggest that making accurate information available is crucial, but not sufficient, for closing partisan gaps. Given the difficulty of relying on individual rationality and widely available information to align individual behaviors for the public good, we recognize the need for policy interventions.

First, our results suggest that interventions that actively disseminate accurate information and encourage information consumption during public health crises, especially among individuals with high health-risks, are needed. Second, and also keeping in mind the fact that times in which mask-wearing mandates were common correspond to erosion of partisan gaps in mask-wearing, and times in which social mobility was restricted (early April) correspond to lower levels of partisan gaps in mobility and self-reported socialization, our results suggest that mandates play an important role in eliminating response differences in politicized public health crises.

### 4.2 Limitations and future directions

We recognize that survey results about a politicized issue are inherently vulnerable to social desirability bias in self-reported behaviors (see, for example, [26]). Our survey design cannot rule out the possibility that Democrats may over-report and Republicans may under-report protective behaviors to signal their political identity. However, the mobility gaps we document are reassuring external evidence that partisan differences in the self-reported survey responses are consistent partisan gaps in the observed discretionary mobility.

In this work, we have argued for the importance of closing the partisan gaps in beliefs and attitudes through information. This recommendation rests on a common assumption made in the economics literature that beliefs and attitudes shape behaviors, which is supported by the positive correlation between risk beliefs and actions in our study, as well as earlier work documenting the predictive power of partisan attitudes on individual actions (e.g., [27]) and the causal impact of political slant in news on voting behavior (e.g., [28, 29]). However, whether partisan perceptions cause differential personal choices is unclear (e.g., [30]). An important avenue for future work is to measure the causal impact of beliefs motivated by political identity on individuals’ health-related choices. If partisan gaps in beliefs indeed shape individual actions, then policies affecting news consumption and, consequently, beliefs would be effective.

Another important avenue for future work is to identify interventions that motivate individuals to consume news about politicized issues. While the literature on partisan news consumption and its impact on polarization is rich (e.g., [31–33]), the literature does not provide solutions to motivate individuals to consume news more broadly that have shown to be effective in the field. Having such a mechanism during the pandemic could have saved lives.

## Supporting information

S1 Appendix SafeGraph data: Robustness analyses. (PDF)

S2 Appendix Survey data: Summary statistics and additional results. (PDF)

S3 Appendix Survey: Consent and questions. (PDF)

S1 File Inclusivity in global research. (DOCX)
