# Supplementary material for: A tale of two pandemics: The enduring partisan differences in actions, attitudes, and beliefs during the coronavirus pandemic
Source: PLoS One. 2023 Oct 25;18(10):e0287018. doi: 10.1371/journal.pone.0287018 (PMC10599506; doi:10.1371/journal.pone.0287018)
Supplement: S1 Appendix — (PDF) [file pone.0287018.s001.pdf]

S1 SafeGraph Data: Robustness Analyses

We conduct two sets of robustness analyses to show our results in Section 3.1 are robust to different adjustment methods and control variables.

Adding Occupational Controls

We noted in the main manuscript that the representation of Democrats and Republicans may systematically differ across occupations. Since workers in some occupations, most notably front-line ones, may have less flexibility to work from home, such differences may contribute to the partisan gaps we observe in mobility for work purposes, especially early on in the pandemic. To fully account for these differences, we would need individual-level data, which is not possible given the anonymity of Safegraph data. Instead, we obtain county-level occupational share data from the United States Census Bureau's American Community Survey 5-year Estimates (5-year ACS) for 2016-2019. Table S1.1 provides average shares of occupations across the 3,110 counties in our data, and the raw correlation between the shares of these occupations and the Democratic vote share in a county.

Table S1.1. County-level Occupation Shares and Correlation with Democratic Voter Shares

Occupations	Average Share	Corr. with Democrat Share
Management, Business, And Financial	.16	.003 (.006)
Computer, Engineering, And Science	.04	.064 (.002)
Education, Legal, Community Svc., Arts, And Media	.09	.056 (.003)
Healthcare Practitioners And Technical	.06	.007 (.002)
Healthcare Support	.02	.005 (.001)
Protective Service	.03	.017 (.002)
Food Preparation And Serving Related	.03	.023 (.002)
Personal Care And Service	.03	.016 (.002)
Building And Grounds Cleaning And Maintenance	.02	.012 (.001)
Sales And Office	.21	.035 (.004)
Natural Resources, Construction, And Maintenance	.17	-.143 (.005)
Production, Transportation, And Material Moving	.14	-.095 (.008)

Notes: The first column reports the average share of each occupation type across counties. The second column reports coefficients (and associated standard errors in parantheses) obtained from regressing the county occupation shares onto county democratic voter shares.

We re-estimate regression equation (1), including time-varying impact of county shares of occupations in various categories as listed in Table S1.1. The results are plotted in Figure S1.1. The partisan gaps in mobility associated with work during the early phase of the pandemic are attenuated, as expected from the conjecture that there might be an over-representation of Democrats in front-line occupations. All general trends remain the same.

Fig S1.1. Mobility Differences, SafeGraph Data, Controlling for Occupation Shares in Each County

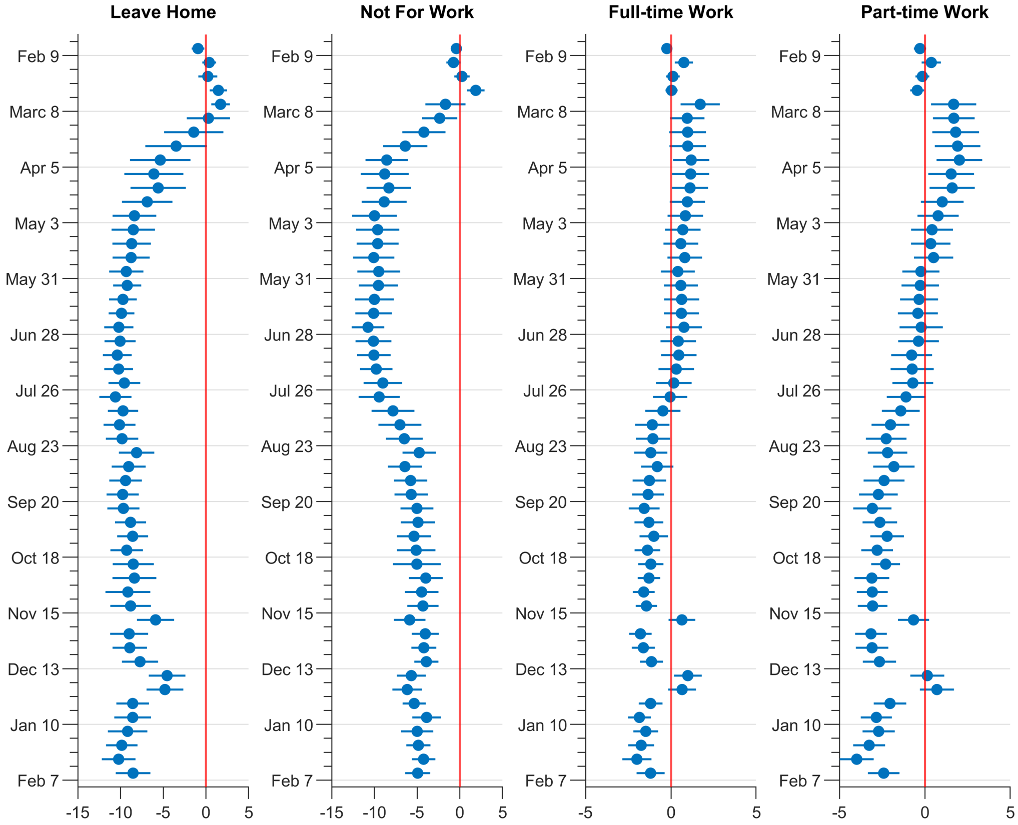

Notes: This figure plots the estimated coefficient $\beta_{w(t)}$ in equation (1) and the corresponding 95% confidence interval where the dependent variable is indicated at the top of each panel, and additional occupation share controls are included in the regression. The week of February 1, 2020 is taken as the baseline $t = 0$. The y-axis markers indicate the beginning date of the week for which the coefficients are reported. Observations are weighted by the number of candidate devices in the county, and standard errors clustered at the county level.

Different Adjustment Methods

SafeGraph reports the number of devices that "pinged" during a given day (active device count). In the main text, we measure the share of devices that remain at home as the unadjusted share $s_{\text{at-home}} = 100 \times$ (completely-at-home devices/active devices). Safegraph notes that the number of completely-at-home devices could be under-reported due to a sampling bias. [25] reports: "GPS data from smartphones is often subject to a sampling bias in favor of devices that are changing locations (i.e., moving). Collecting GPS data is battery-intensive, and software applications sometimes implement GPS data collection methods that depend on the movement of the device, rather than a fixed time interval. This represents a sampling bias in favor of detecting devices that are moving." SafeGraph therefore also reports the number of all devices in its sample during a month, regardless of whether it saw any activity for them on a specific day within the month (candidate device count). However, it is not clear whether this number reflects the number of devices that could have been reporting on a given day, since Safegraph's sample of phones dynamically evolves over time.

We explore the robustness of our results to the following two alternative approaches of calculating shares of devices showing different types of activity:

1. **Using the maximum number of devices that pinged in a week as the denominator and basis for adjustment.** In this approach, we calculate the largest number of active devices each week for each county ("max active device"), assume it to be the latent true number of active devices for that week. As a result, the number of at-home devices are adjusted by the difference (max active devices - active devices) to account for potentially latent at-home devices that did not ping. We define the dependent variables as: $s_{\text{part-time}} = 100 \times (\frac{\text{part-time work devices}}{\text{max active devices}})$, $s_{\text{full-time}} = 100 \times (\frac{\text{full-time work devices}}{\text{max active devices}})$, $s_{\text{at-home}} = 100 \times (\frac{\text{completely-at-home devices+(max active devices - active devices)\}}}{\text{max active devices}})$, $s_{\text{leave-home}} = 100 - s_{\text{at-home}}$, and $s_{\text{not-work}} = 100 - s_{\text{at-home}} - s_{\text{part-time}} - s_{\text{full-time}}$. The upper part of Figure S1.2 reports the estimates.

2. **Using candidate devices as the denominator and basis for adjustment.** In this approach, we assume the number of candidate devices to be the latent true number of active devices for each day, and assume that any non-active device was at-home. As a result, the number of at-home devices are adjusted by the difference ($candidatedevices - activedevices$) to account for potentially latent at-home devices that did not ping. We define the dependent variables as: $s_{\text{part-time}} = 100 \times (\frac{\text{part-time work devices}}{\text{candidate devices}})$, $s_{\text{full-time}} = 100 \times (\frac{\text{full-time work devices}}{\text{candidate devices}})$, $s_{\text{leave-home}} = 100 - s_{\text{at-home}}$, where $s_{\text{at-home}} = 100 \times (\frac{\text{completely-at-home devices+(candidate devices - active devices)\}}}{\text{candidate devices}})$, and $s_{\text{not-work}} = 100 - s_{\text{at-home}} - s_{\text{part-time}} - s_{\text{full-time}}$. The lower part of Figure S1.2 reports the estimates.

Although the results change across specifications, the general message of a persistent partisan gap in social mobility remains the same.

Fig S1.2. Partisan Gaps in Mobility: Alternative Adjustment Methods

(A) Alternative Adjustment Method (1)

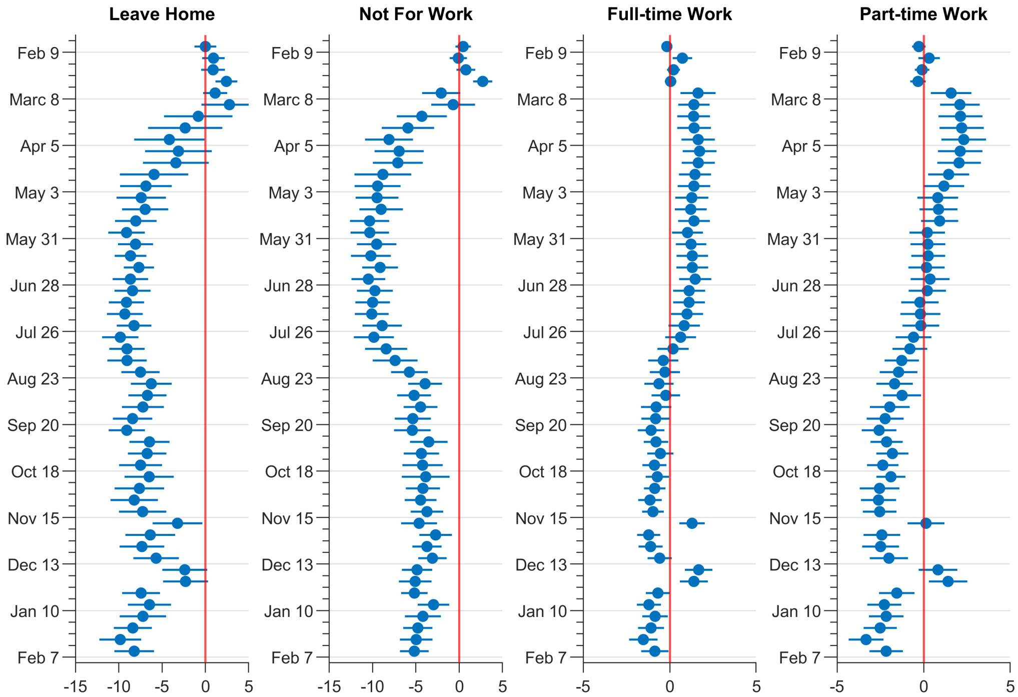

(B) Alternative Adjustment Method (2)

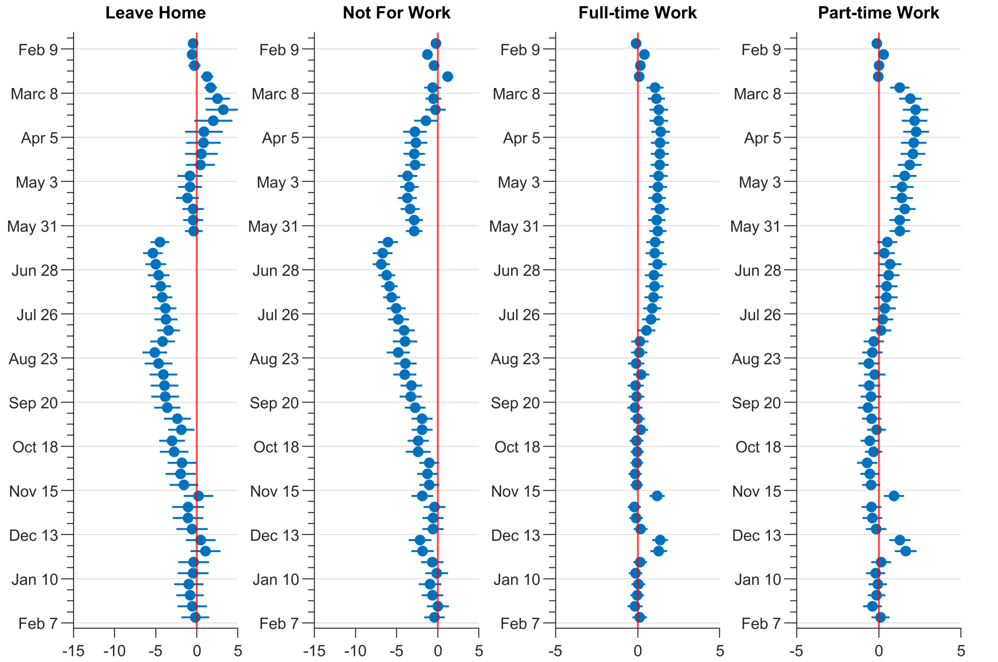

Notes: This figure plots the estimated coefficient $\beta_{w(t)}$ in equation (1) and the corresponding 95% confidence interval where the dependent variable is indicated at the top of each panel. The week of February 1, 2020 is taken as the baseline $t = 0$. The y-axis markers indicate the beginning date of the week for which the coefficients are reported. Observations are weighted by the number of candidate devices in the county, and standard errors clustered at the county level.
