# Supplementary material for: A tale of two pandemics: The enduring partisan differences in actions, attitudes, and beliefs during the coronavirus pandemic
Source: PLoS One. 2023 Oct 25;18(10):e0287018. doi: 10.1371/journal.pone.0287018 (PMC10599506; doi:10.1371/journal.pone.0287018)
Supplement: S2 Appendix — (PDF) [file pone.0287018.s002.pdf]

S2 Survey Data: Summary Statistics and Additional Results

Summary Statistics

Table S2.1. Representativeness of Survey Respondents

	Mean	National Mean[a]
Female	0.51	0.51
Age: 18-24	0.13	0.12
Age: 25-34	0.18	0.18
Age: 35-44	0.19	0.16
Age: 45-54	0.15	0.16
Age: 55-64	0.19	0.17
Age: 65+	0.17	0.21
Asian	0.05	0.06
Black	0.10	0.13
White	0.70	0.77
Region - Midwest	0.21	0.21
Region - Northeast	0.19	0.17
Region - South	0.38	0.38
Region - West	0.22	0.24

[a]Share of persons age 18+ years.

Lucid provided demographic variables of respondents. Table S2.1 compares the shares of respondents with a certain gender, age, race, or live in a certain region with the corresponding national shares. We obtain the national shares from Census "Estimates of the Total Resident Population and Resident Population Age 18 Years and Older for the United States, States, and Puerto Rico: July 1, 2019," and Census "Annual Estimates of the Resident Population by Sex, Age, Race, and Hispanic Origin for the United States: April 1, 2010 to July 1, 2019.". Table S2.1 shows that our survey sample is representative.

Table S2.2 reports the summary statistics of the categorical control variables we construct and employ in our regressions. We indicate an individual as a Democrat (Republican) if they chose of the following responses to the political affiliation question: "Strong Democrat (Republican)," "Not very strong Democrat (Republican)," "Independent Democrat (Republican)" or "Other - leaning Democrat (Republican)." We indicate an individual as Independent if they chose of the following responses to the political affiliation question: "Independent - neither" or "Other - neither." Alternative specifications of this variable do not substantively change our results.

While Table S2.2 reports the overall summary statistics of the sample, Figure S2.1 gives the average responses over time.

Finally, Table S2.3 reports summary statistics about news consumption and information attention levels by political affiliations.

Table S2.2. Summary Statistics on Demographics of Survey Respondents (N=13,334)

	Mean	Standard Deviation
Female	0.51	0.50
Age: 18-24	0.13	0.33
Age: 25-34	0.18	0.38
Age: 35-44	0.19	0.36
Age: 45-54	0.15	0.39
Age: 55-64	0.19	0.38
Age: 65+	0.17	0.33
Asian	0.05	0.21
Black	0.10	0.30
White	0.70	0.46
Other non-white	0.16	0.36
Democrat	0.46	0.50
Republican	0.39	0.49
Independent	0.15	0.36
High school degree	0.22	0.41
Bachelor's degree	0.34	0.47
Post-graduate degree	0.18	0.38
Education level missing	0.005	0.07
Household income $< 30k$	0.33	0.47
Household income $\in [30k, 75k)$	0.35	0.48
Household income $\geq 75k$	0.28	0.45
Household income missing	0.05	0.21

Fig S2.1. Summary Statistics of Dependent Variables: Survey

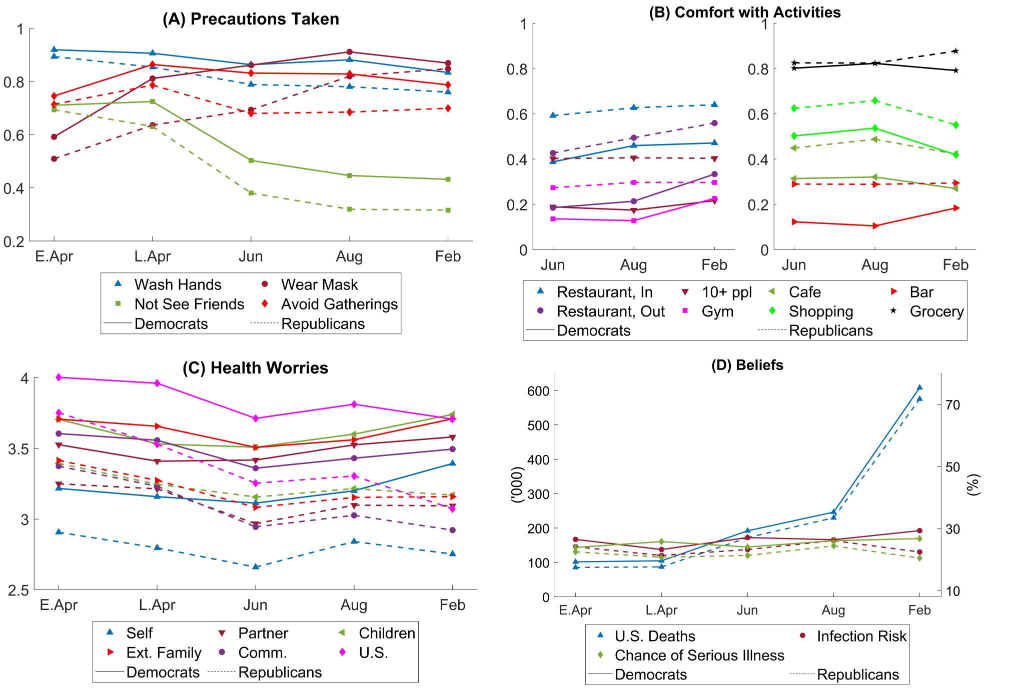

Notes: This figure plots average responses by political affiliations over time. Markers connected by a solid line indicate Democrats while markers connected by a dash line Republicans. The x-axis indicates the period. In Panel A, the y-axis gives the fraction of respondents who have taken an action indicated in the legend. The actions are "Wash Hands"–wash hands more often; "Wear Mask"– wear a mask when out and about; "Not See Friends"– do not meet any friends or extended family; "Avoid Gatherings"– avoid public transportation and large gatherings. In Panel B, the y-axis gives the fraction of respondents who feel comfortable with an activity indicated in the legend. The activities are "Restaurant, In"– eat in a restaurant with indoor seating; "Restaurant, Out"– eat in a restaurant with outdoor seating; "10+ ppl"– be part of a gathering with more than 10 people; "Cafe"– go to a coffee shop; "Bar"–go to a bar; "Gym"–go to the gym; "Shopping"– go shopping for non-grocery items; "Grocery"– go grocery shopping. In Panel C, the y-axis is how much respondents worry (on a scale of 1 to 5, larger numbers corresponding to more worry) about the health of people indicated in the legend. The groups of people are "Self"– respondent herself; "Partner"– respondent's partner; "Children"– respondent's kids; "Ext. Family"– respondent's extended family; "Community"– members of the respondent's community; "U.S."– all people in the U.S. In Panel D, the y-axis is respondents' prediction on the outcomes indicated in the legend. Outcomes over which expectations are elicited are "U.S. Deaths"– total number of deaths in the U.S. by a target date (in thousands); "Chance of Infection"– chances that the respondent will get infected with the coronavirus in the next three month (in %); "Chance of Serious Illness"– chances that the respondent will have serious symptoms should she get infected (in %).

Table S2.3. Summary Statistics of News Exposure and Attention

	All	Democrats	Other	Republicans
Generally consume				
ABC	.36	.42	.28	.31
CNN	.38	.53	.27	.24
Fox News	.50	.46	.34	.59
HuffPost	.07	.10	.03	.04
NBC/MSNBC	.30	.40	.21	.21
NYT	.20	.27	.11	.14
NPR	.11	.16	.06	.07
Wall Street Journal	.11	.12	.07	.11
Washington Post	.14	.20	.08	.09
Regarding the pandemic, pay attention to				
Friends	2.87	2.88	2.75	2.88
	(12,215)	(5,665)	(1,768)	(4,782)
Family	3.19	3.20	3.06	3.23
	(12,353)	(5,714)	(1,826)	(4,813)
Twitter	1.97	1.99	1.68	2.06
	(7,383)	(3,361)	(1,048)	(2,974)
Facebook	2.07	2.02	1.86	2.21
	(8,468)	(3,798)	(1,211)	(3,459)
Twitter or Facebook	2.22	2.50	1.91	1.92
	(2,465)	(1,278)	(345)	(842)
Pastor	2.47	2.38	2.23	2.64
	(9,822)	(4,396)	(1,352)	(4,074)
CDC	3.69	4.02	3.28	3.45
	(12,415)	(5,772)	(1,812)	(4,831)
Scientists	3.72	4.04	3.27	3.50
	(12,345)	(5,724)	(1,809)	(4,821)
Governor	3.13	3.26	2.66	3.15
	(12,442)	(5,771)	(1,810)	(4,861)
President	2.76	2.36	2.43	3.35
	(12,387)	(5,735)	(1,799)	(4,853)

Notes: The upper panel reports the percentage (among all 13,334) of respondents who consume a particular news outlet. The bottom panel reports the average rating of the degree of attention individuals pay to different sources of information (ranging from 1 – Not at all to 5 – Very much so), if they have indicated that source as applicable. The number of individuals who indicate the source as applicable is reported in parentheses. The attention question was not included in the first wave, therefore 12,852 respondents received this question.

Additional Results

We present three additional results using the survey data. First, we show that our results are robust to considering the Independent - Republican differences instead of the Democratic - Republican differences. These results are presented in Fig S2.2.

Second, in addition to health worries, the survey also asked respondents about how much they feel worried about the economic well-being of themselves, their partner, their children, their extended family, their community, and the people in the U.S. In Figure S2.3, while Panel A plots the summary statistics for these economic worries, Panel B plots the estimated α_τ's from regression equation (2) depicting Democrat vs. Republican differences.

Third, we show that there are significant and stable associations between individuals' individual risk beliefs and their actions and attitudes. Fig S2.4 shows a positive association between beliefs regarding the severity of disease and whether or not a person engages in a given protective behavior and a negative correlation between disease severity beliefs and feeling comfortable engaging in economic activity. For example, one standard deviation increase in the perceived chance of serious illness is associated with a 2.9% - 8.5% increase in self-reported refrain from seeing friends across the 10-month span, and a 5%-7.6% decrease in whether the individual is comfortable with dining indoors. Similarly, Fig S2.5 and Fig S2.6 show that there are positive correlations between actions and attitudes with infection risk beliefs and with death toll expectations as well.

Moreover, the associations between risk perceptions and protective behaviors and comfort with economic activities are stable over time. An exception is the correlation between beliefs and wearing masks. In the early periods of the pandemic, the correlation is substantial, but as mask mandates are instituted, this behavior-belief correlation disappears. Earlier work has also documented correlations between beliefs and attitudes. In particular, [16] document an association between infection risk beliefs and economic activity at the beginning of the pandemic. [1] document a positive correlation between death toll expectations and self-reported social distancing. Here, we focus on severity beliefs instead of infection risks beliefs because the latter may be a result of individual actions (e.g., one thinks they are less likely to be infected because one avoids socialization). We also present evidence of a sustained association over time. Therefore, our results should be interpreted as extending prior evidence both temporally, and using a different risk belief that is both individually relevant and less susceptible to reverse causation.

Fig S2.2. Partisan Gaps in Actions, Attitudes, Worries, Beliefs: Independents vs. Republicans

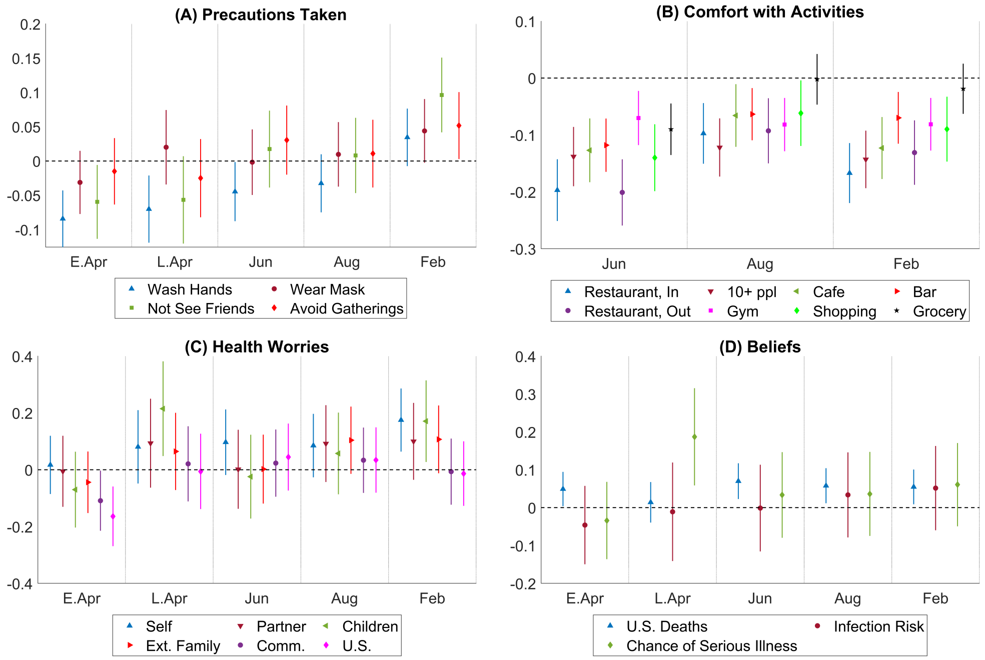

Notes: This figure plots the estimated Independent - Republican partisan gaps obtained from the estimates of α_τ in equation (2) and the corresponding 95% confidence intervals. The x-axis indicates the period τ. In Panel A, a positive estimate means that, *ceteris paribus*, independent respondents are more likely than Republican respondents to have taken an action indicated in the legend. The actions studied are “Wash Hands”–wash hands more often; “Wear Mask”– wear a mask when out and about; “Not See Friends”– do not meet any friends or extended family; “Avoid Gatherings”– avoid public transportation and large gatherings. In Panel B, a positive estimate means that, *ceteris paribus*, Independent respondents are more likely than Republican respondents to feel comfortable with an activity indicated in the legend. Activities studied are “Restaurant, In”– eat in a restaurant with indoor seating; “Restaurant, Out”– eat in a restaurant with outdoor seating; “10+ ppl”– be part of a gathering with more than 10 people; “Cafe”– go to a coffee shop; “Bar”– go to a bar; “Gym”– go to a gym; “Shopping”– go shopping for non-grocery items; “Grocery”– go grocery shopping. In Panel C, a positive estimate means that, *ceteris paribus*, independent respondents worry more about the health of the group of people indicated in the legend. The groups of people are “Self”– respondent herself; “Partner”– respondent’s partner; “Children”– respondent’s kids; “Ext. Family”– respondent’s extended family; “Comm.”– members of the respondent’s community; “U.S.”– all people in the U.S. In Panel D, a positive estimate means that, *ceteris paribus*, independent respondents predict a larger number on the outcomes indicated in the legend. Outcomes over which expectations are elicited are “U.S. Deaths”– total number of deaths in the U.S. by a target date; “Chance of Infection”– chances that the respondent will get infected with the coronavirus in the next three months; “Chance of Serious Illness”– chances that the respondent will have serious symptoms should she get infected.

Fig S2.3. Partisan Gaps in Economic Worries

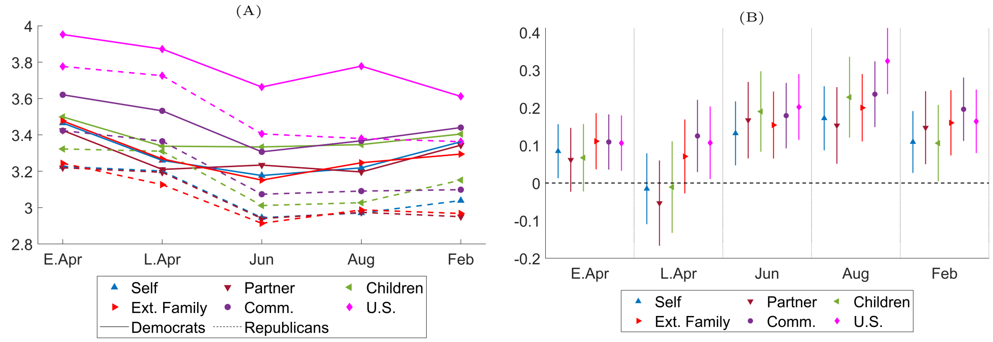

Notes: Panel A plots average values of economic worries across the respondents. Panel B plots the estimated Democrat - Republican partisan gaps obtained from the estimates of α_τ in equation (2) and the corresponding 95% confidence intervals. The x-axis indicates the period τ. In Panel B, a positive estimate means that, *ceteris paribus*, Democrat respondents are more worried than Republican respondents about the group of people indicated in the legend. The groups of people are "Self"– respondent herself; "Partner"– respondent's partner; "Children"– respondent's kids; "Ext. Family"– respondent's extended family; "Comm."– members of the respondent's community; "U.S."– all people in the U.S.

Fig S2.4. Correlation between Outcome Severity Beliefs and Protective Behaviors and Attitudes towards Economic Activity

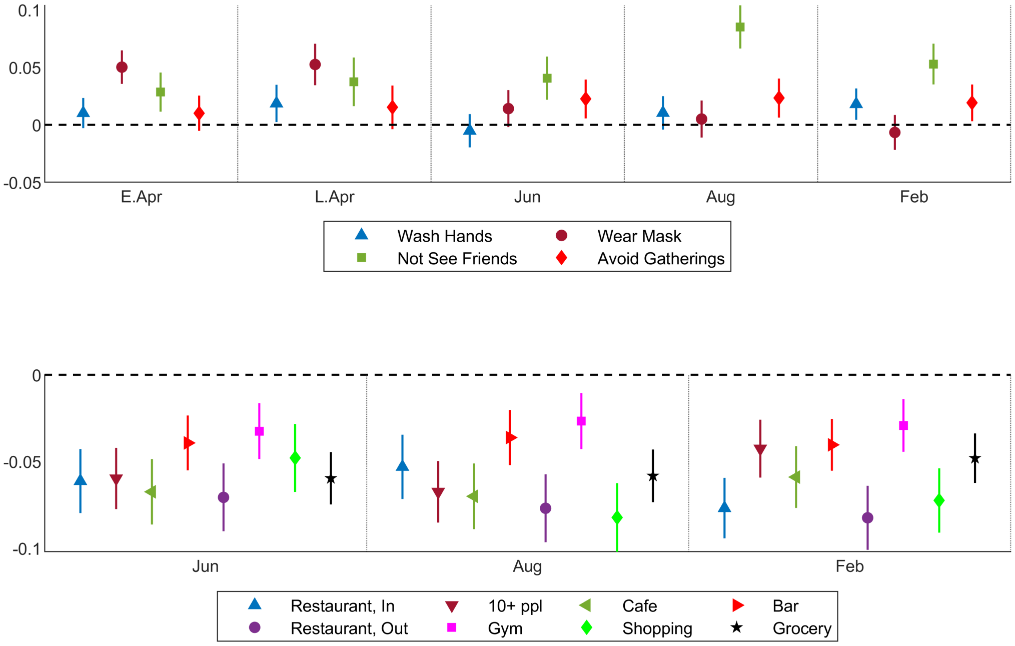

Notes: This figure plots the correlation between an individual's belief about her chance of getting serious symptoms should she gets infected and her likelihood of taking a certain protective action (in the upper panel) and feeling comfortable with a certain economic activity (in the lower panel). The x-axis indicates the period. In the upper panel, the actions studied are "Wash Hands"–wash hands more often; "Wear Mask"– wear a mask when out and about; "Not See Friends"– do not meet any of friends or extended family; "Avoid Gatherings"– avoid public transportation and large gatherings. In the lower panel, activities studied are "Restaurant, In"– eat in a restaurant with indoor seating; "Restaurant, Out"– eat in a restaurant with outdoor seating; "10+ ppl"– be part of a gathering with more than 10 people; "Cafe"– go to a coffee shop; "Bar"– go to a bar; "Gym"– go to a gym; "Shopping"– go shopping for non-grocery items; "Grocery"– go grocery shopping.

Fig S2.5. Correlation between Infection Risk Beliefs and Protective Behaviors and Attitudes towards Economic Activity

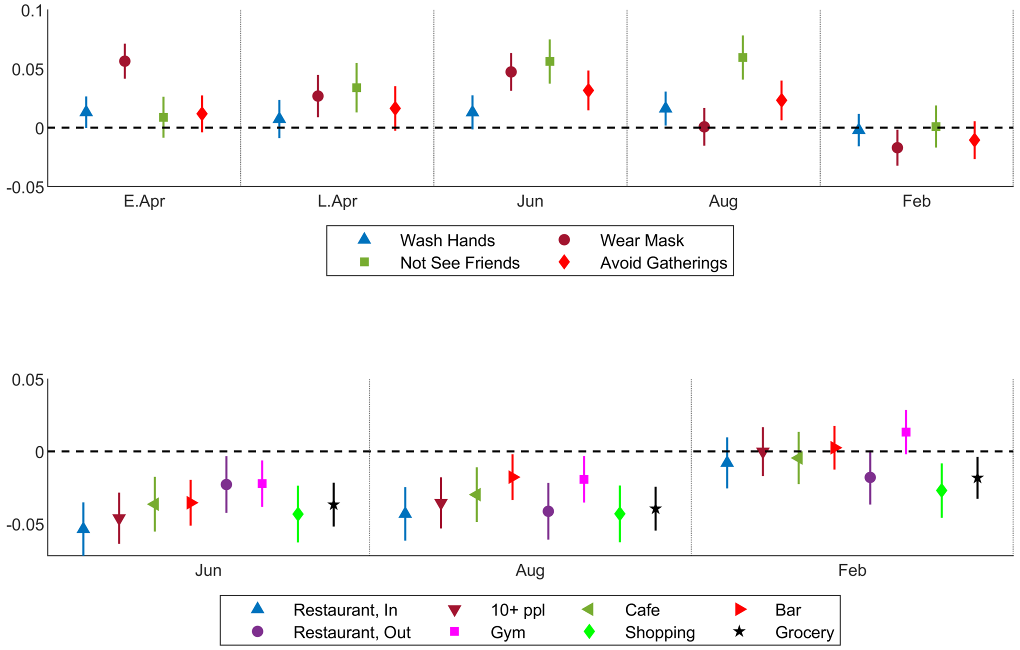

Notes: This figure plots the correlation between an individual's belief about her chance of getting infected in the next three months and her likelihood of taking a certain protective action (in the upper panel) and feeling comfortable with a certain economic activity (in the lower panel). The x-axis indicates the period. In the upper panel, the actions studied are "Wash Hands"–wash hands more often; "Wear Mask"– wear a mask when out and about; "Not See Friends"– do not meet any of friends or extended family; "Avoid Gatherings"– avoid public transportation and large gatherings. In the lower panel, activities studied are "Restaurant, In"– eat in a restaurant with indoor seating; "Restaurant, Out"– eat in a restaurant with outdoor seating; "10+ ppl"– be part of a gathering with more than 10 people; "Cafe"– go to a coffee shop; "Bar"– go to a bar; "Gym"– go to a gym; "Shopping"– go shopping for non-grocery items; "Grocery"– go grocery shopping.

Fig S2.6. Correlation between U.S Total Deaths and Protective Behaviors and Attitudes towards Economic Activity

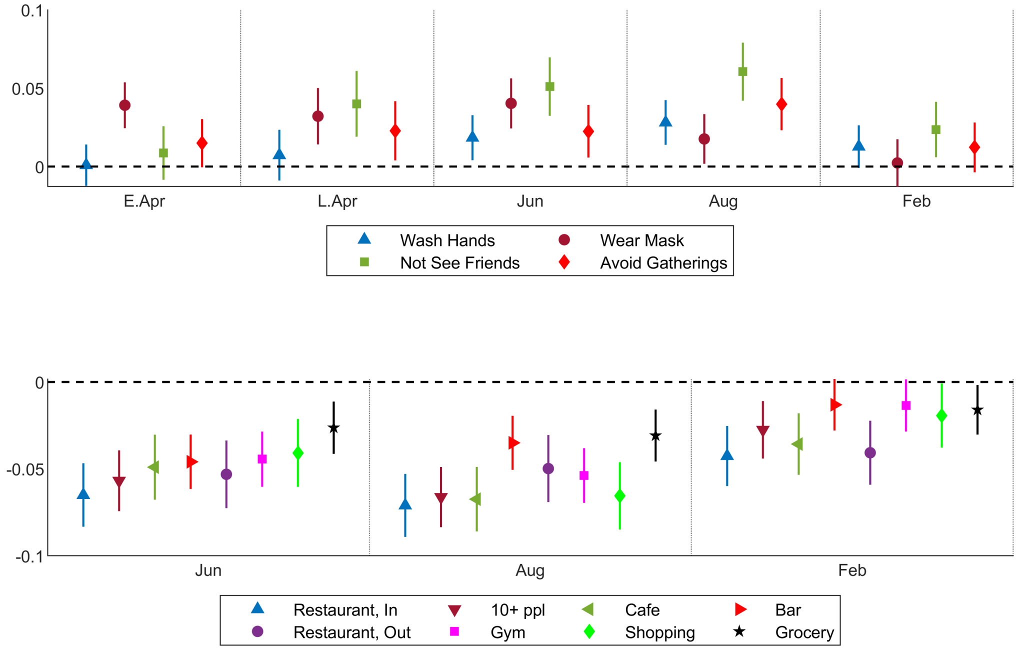

Notes: This figure plots the correlation between an individual's prediction about the future death toll of the pandemic in the U.S. and her likelihood of taking a certain protective action (in the upper panel) and feeling comfortable with a certain economic activity (in the lower panel). The x-axis indicates the period. In the upper panel, the actions studied are "Wash Hands"–wash hands more often; "Wear Mask"– wear a mask when out and about; "Not See Friends"– do not meet any of friends or extended family; "Avoid Gatherings"– avoid public transportation and large gatherings. In the lower panel, activities studied are "Restaurant, In"– eat in a restaurant with indoor seating; "Restaurant, Out"– eat in a restaurant with outdoor seating; "10+ ppl"– be part of a gathering with more than 10 people; "Cafe"– go to a coffee shop; "Bar"– go to a bar; "Gym"– go to a gym; "Shopping"– go shopping for non-grocery items; "Grocery"– go grocery shopping.
