# Supplementary material for: A tale of two pandemics: The enduring partisan differences in actions, attitudes, and beliefs during the coronavirus pandemic
Source: PLoS One. 2023 Oct 25;18(10):e0287018. doi: 10.1371/journal.pone.0287018 (PMC10599506; doi:10.1371/journal.pone.0287018)
Supplement: S3 Appendix — (PDF) [file pone.0287018.s003.pdf]

S3 Survey: Consent and Questions

Survey Consent

The survey starts with the following consent form:

You are invited to participate in a research study about COVID-19. This is a 15-minute long survey that will ask about your perceptions, expectations and feelings about the disease, its effects on you and on our nation. If you agree to be part of the research study, you will be asked to provide your opinions on policies, risks, and will be asked to answer questions related to your current situation. Please pay attention to all questions. We will include several attention checks.

Benefits of the research to the public stem from your participation and honest answers. Using this survey data, we hope to be able to provide guidelines for assessing and responding to differences across communities. Risks and discomforts: Thinking about COVID-19 and its impact may induce negative emotions, like anxiety or fear. These risks and discomforts are minimal for most people.

Participating in this study is completely voluntary. Even if you decide to participate now, you may change your mind and stop at any time. You may choose not to continue with the survey at any time and for any reason.

There is no deception or false information in this survey.

We will protect the confidentiality of your research records by not publishing any information that may identify you. Information collected in this project may be shared with other researchers, and may be connected to other aggregate datasets at the county level. We will not share any information that could identify you. All results will be reported in aggregate.

Principal Investigator: Yesim Orhun, Associate Professor, University of Michigan. If you have questions about this research study, please contact Prof. Yesim Orhun by emailing aorhun@umich.edu. The University of Michigan Institutional Review Board Health Sciences and Behavioral Sciences has determined that this study is exempt from IRB oversight.

By clicking to proceed, you are confirming that you read this page and are providing consent to participate.

Survey Questions

In what follows, survey questions are in normal font while our notes are in italic.

Risk Tolerance

- Thinking about yourself, in general, how willing or unwilling are you to take risks? Please use the scale below, ranging from 0 to 10, where 0 means “completely unwilling to take risks” and a 10 means you are “very willing to take risks.” You can also use any number between 0 and 10 to indicate where you fall on the scale. [Scale: 1 to 10, choose one.]

Employment and Economic Impact, Zip code

- What’s your current employment status? [Choose one: Employed full time; Employed part time; Furloughed; Unemployed (before the coronavirus); Unemployed (after the coronavirus); Retired; Student; Prefer not to answer.]
- Please think of everyone in your household who was earning an income before the coronavirus crisis. What was the economic impact of the coronavirus situation on the income of your household? [Choose one: Greatly negative; Very negative;

Somewhat negative; No change; Somewhat positive; Very positive; Greatly positive.]

- What is your zip code? [Fill in.]

Personal Experience with COVID-19

- How has the health of the community you live in been impacted by the coronavirus? [Choose one: So far, we don't have any cases or deaths; We have only a few cases and no deaths; We have a moderate number of cases, but no deaths; We have a moderate number of cases, and at least one death; We have a lot of cases, but no deaths; We have a lot of cases, and at least one death; We have a lot of cases and a lot of deaths.]
- Have you been infected with the coronavirus? [Choose one: Yes, I tested positive; No, I tested negative; Probably yes, but I did not get tested; Probably not, but I did not get tested.]
- Do you have friends or family members who have been severely ill with the coronavirus? [Choose one: Yes; We suspect it was due to coronavirus, but we don't know for sure; No.]
- Do you have friends or family members who have been hospitalized or have died due to a coronavirus infection? [Choose one: Yes; We suspect it was due to coronavirus, but we don't know for sure; No.]
- Do you have any health conditions you know of that put you in the high-risk category for serious complications arising from a coronavirus infection? [Choose one: Definitely yes; Most likely yes; I am not sure; Most likely not; Definitely not.]

Attention Check, Screening

- Please think of everyone in your community who has been affected by the coronavirus crisis. It is important that you pay attention to this survey. Please check greatly positive below. [Choose one: Greatly negative; Very negative; Somewhat negative; No change; Somewhat positive; Very positive; Greatly positive.] *Those who failed this attention check were not allowed to proceed with the rest of the survey and their survey responses were not recorded.*

Mandates/Restrictions in Place

- (*asked only in April*) Has your state introduced any social distancing measures? Please click all that apply in your state at this time. [Choose all that apply: No social distancing measures at this time; Schools are closed; Congregating at churches is not allowed; Restaurants/bars closed, except for take-out/delivery; Gyms are closed; Workers cannot travel to work at non-essential businesses; Gatherings of 50+ people are forbidden, less than 50 is ok; Gatherings of 10+ people are forbidden, less than 10 is ok; All social gatherings are forbidden; Stay-at-home order.]
- (*asked in June, August, and February*) What are the current COVID-19 related measures and restrictions that apply where you live at this time? [No measures at this time; Schools are closed; Congregating at churches is not allowed; Restaurants/bars closed, except for take-out/delivery; Gyms are closed; Many workplaces are not allowed to have employees or customers; Masks are required in most indoor public places; Masks are recommended in most indoor public places,

but not required; All social gatherings are forbidden; Gatherings of 50+ people are forbidden, less than 50 is ok; Gatherings of 10+ people are forbidden, less than 10 is ok; Stay-at-home order.]

Protective Behaviors

- Which of the following changes have you made to protect yourself from the coronavirus infection? Please click all that apply. [Choose all that apply: I did not make any changes; Wash hands more frequently; Canceled travel plans; Avoid large gatherings; Work from home; Wear gloves when I go shopping; Wipe down groceries after I bring them home; Wear a mask when I am out and about; Do not meet any of my friends or extended family in-person; Avoid all public places and self-isolate at home; I made other changes: (fill in).]

Comfort with Economic Activities

- (*asked in June, August, and February*) At this time, which of the following activities do you feel comfortable engaging in? [Yes/No options were available for the following list of activities: Eat at a restaurant (outside seating); Eat at a restaurant (inside seating); Go into a coffee shop; Go into a bar/pub; Use public restrooms; Go grocery shopping; Go shopping for non-food items (at the mall, hardware store, etc.); Go to the beach; Go to the gym or other sports facility; Be part of a gathering with more than 10 people (church, school, meetings, work, etc.).]

Worries

- How worried are you feeling for the health of the following people? If you don't have the people mentioned in some statements (partner, kids, extended family), please click "Not Applicable". [Groups of people: My own health; My partner's health; My kids' health; My extended family's health; The health of doctors and nurses in my community; The health of other members of my community; The health of people in big cities like New York, Seattle, Detroit, San Francisco; The health of all the people in the US.] [Choose one: Not at all worried, slightly worried, moderately worried, very worried, extremely worried. Also an option: not applicable.]

- How worried are you feeling for the economic well-being of the following people? [Same options and groups as above.]

Beliefs About Infection Risk

As of April 20, 2020, CDC (Centers for Disease Control and Prevention) is reporting 776,093 confirmed coronavirus cases and 41,758 deaths in the U.S. Many cases go undetected.[1] Of course, infection rates depend on the community and the protection measures each person can take. Assuming that the social distancing policies and your personal efforts stay the same, what are the chances that you will get infected with the coronavirus in the next three months? [Choose one: 0% chance; 1-10% chance; 11-20% chance; 21-30% chance; 31-40% chance; 41-50% chance; 51-60% chance; 61-70% chance; 71-80% chance; 81-90% chance; 91-100% chance.]

[1] The date and these two numbers were updated to the applicable information of two days prior to the survey time.

Beliefs About the Effectiveness of State Restrictions and Own Precautions in Reducing Infection Risk (*asked only in April*)

The next two questions embedded responses from previous questions. If the respondent indicated that their state has not introduced any social distancing measures, or that they have not taken any precautions, the relevant question was not displayed to the respondent.

- Earlier, the survey asked about the changes you personally made to protect yourself from the coronavirus infection. You indicated that you [all precautions that the respondent indicated as having taken]. Assuming that the state policies stay the same, what do you think your chances of becoming infected with coronavirus in the next three months would be if you did not make these changes? [Choose one: Same chance; 5% higher chance; 10% higher chance; 15% higher chance; 20% higher chance; 25% higher chance; 30% higher chance; 40% higher chance; 50% higher chance; My chance of being infected would increase by more than 50%.]

- Imagine that you could still take the same measures you personally took to lower your chances of being infected with coronavirus, but your state had not introduced any social distancing measures. Assuming your personal efforts stay the same, what do you think your chances of becoming infected with coronavirus in the next three months would be if your state did not have social distancing measures? [Choose one: Same chance; 5% higher chance; 10% higher chance; 15% higher chance; 20% higher chance; 25% higher chance; 30% higher chance; 40% higher chance; 50% higher chance; My chance of being infected would increase by more than 50%.]

Beliefs About Health Outcomes Conditional on Being Infected

- According to the CDC (Centers for Disease Control and Prevention) report, about 7% of people diagnosed with the coronavirus are hospitalized, but do not need intensive care. About 1.5% of people are hospitalized and need intensive care. It is also suspected that a large percentage of people are symptom-free and/or have mild versions of the disease.

  Most importantly, the chances are person-specific. The progression of the disease can be very different based on your age, health, pre-existing condition, living conditions, how much of the virus you are exposed to, etc. Although it's hard to know without data, you probably have a better understanding of your situation than anyone else. Therefore, we ask you to predict how the coronavirus is likely to affect you, should you get infected:

  Please make sure numbers add up to 100. Allocate points according to how big you think your chances are for each possibility. [Chances that I will be symptom-free are: (fill in, numerical); Chances that I will have a mild version of the disease are: (fill in, numerical); Chances that I will have a moderate version (without hospitalization) are: (fill in, numerical); Chances that I will have a severe version that requires hospitalization (but no further interventions) are: (fill in, numerical); Chances that I will have a severe version that requires intensive care at the hospital are: (fill in, numerical).]

Factual Questions About Factors That Influence Health Outcomes

- Quality of care. Communities differ in how well equipped their hospitals are and how much capacity their hospitals have when it comes to coronavirus infections. If

you were to be hospitalized due to coronavirus, what's your expectation of the quality of care you would receive? [Choose one: Top-notch care, no issues; Good care, as usual; Good care, but hospitals would have bed/equipment shortages; OK care, as usual; OK care, but hospitals would have bed/equipment shortages; Not so good care, as usual; Not so good care, and hospitals would have bed/equipment shortages.]

- Pre-existing health conditions. The CDC (Centers for Disease Control and Prevention) released the list of underlying medical conditions that put people of all ages at higher risk for severe illness resulting from the coronavirus infection. We list them below.

  Which of these apply to you? Please click all that apply. [Choose all that apply: Moderate to severe asthma; COPD or other chronic lung disease; Serious heart conditions; Diabetes; Conditions that can cause a person to be immunocompromised, including cancer treatment, smoking, bone marrow or organ transplantation, immune deficiencies, poorly controlled HIV or AIDS, and prolonged use of corticosteroids and other immune weakening medications.; Severe obesity (BMI of 40 or higher); Chronic kidney disease and currently undergoing dialysis; Liver disease; I do not want to answer; None of them apply to me.]

Beliefs About Systemic Health Risk (Total Number of Deaths in the U.S.)

- As of April 20, 2020, CDC (Centers for Disease Control and Prevention) is reporting 41,758 deaths in the US.[2] Assuming the state policies remain the same, how many people do you think will die from a coronavirus infection in the U.S. by July 1, 2020? [Choose one: less than 25,000; 25,000–50,000; 50,000–75,000; 75,000–100,000; 100,000–125,000; 125,000–150,000; 150,000–175,000; 175,000–200,000; 200,000–225,000; 225,000–250,000; 250,000–275,000; 275,000–300,000; 300,000–350,000; more than 350,000.]

Beliefs About Economic Outcomes (*asked only in April*)

- The U.S. gross domestic product (GDP) grew about 2.3% in 2019. Assuming the state policies remain the same, how much GDP growth do you expect in 2020? [Choose one: more than 10% growth; 5%–10% growth; 2.5%–5% growth; 0–2.5% growth; 0% to -2.5% growth (negative growth means contraction); -2.5% to -5% growth (negative growth means contraction); -5% to -10% growth (negative growth means contraction); -10% to -20% growth (negative growth means contraction); -20% to -30% growth (negative growth means contraction); worse than -30% growth (negative growth means contraction).]

- In the last quarter of 2019, unemployment rate in the U.S. was 3.6%. Assuming the state policies remain the same, how much unemployment do you expect in the U.S. by July 1, 2020? [Choose one: less than 3%; 3-5%; 5-10%; 10-15%; 15-20%; 20-25%; 25-30%; more than 30%.]

Beliefs About the Effectiveness of State Policies in Reducing Total Number of Deaths in the U.S. and Their Impact on Economic Outcomes (*asked only in April*)

At this time, the majority of states are implementing some measure of social distancing. Assuming there are no changes to these policies, you expect that by July 1st [reminder

[2] The date and the number of deaths were updated to the applicable information of two days prior to the survey time.

of their previous death prediction] people in the U.S. will die due to coronavirus infections. You also expect [reminder of their previous unemployment prediction] rate of unemployment by July 1st, and [reminder of their previous GPD growth prediction] in GDP in 2020. How would these predictions change if none of the states implemented any social distancing measures?

- Please select the number of U.S. deaths you would expect by July 1st if there were no social distancing measures. [Choose one: less than 25,000; 25,000–50,000; 50,000–75,000; 75,000– 100,000; 100,000–125,000; 125,000–150,000; 150,000–175,000; 175,000–200,000; 200,000–225,000; 225,000–250,000; 250,000–275,000; 275,000–300,000; 300,000–350,000; more than 350,000.]
- Please select the rate of GDP growth you would expect in 2020 if there were no social distancing measures. [Choose one: more than 10% growth; 5%–10% growth; 2.5%–5% growth; 0–2.5% growth; 0% to -2.5% growth (negative growth means contraction); -2.5% to -5% growth (negative growth means contraction); -5% to -10% growth (negative growth means contraction); -10% to -20% growth (negative growth means contraction); -20% to -30% growth (negative growth means contraction); worse than -30% growth (negative growth means contraction).]
- Please select the rate of unemployment you would expect by July 1st if there were no social distancing measures. [Choose one: less than 3%; 3-5%; 5-10%; 10-15%; 15-20%; 20-25%; 25-30%; more than 30%.]

Attitudes Towards Policies (*asked only in April*)

- Policymakers are debating the effectiveness and pros/cons of many options. We listed some of these options below. Which of them do you most agree/disagree with regarding their sensibility at this time, in your community? [Policy recommendations: Washing hands more often, avoiding handshakes; Keeping kids out of school; Not sending people to work, unless they are essential workers; Forbidding all social gatherings; Requiring people to stay at home.] [Choose one: Strongly disagree; Disagree; Somewhat disagree; Neither agree nor disagree; Somewhat agree; Agree; Strongly agree.]

Constraints as Main Drivers of Choices

Everyone has different preferences, risks, constraints in their lives. Earlier in the survey, we asked you what self-protective measures you took, if any. On this page, we ask why you did not choose some of the self-protective actions. These questions are not meant as judgement. They aim to understand what actually drives people's decisions. Please answer them honestly. We want to know more about people's preferences and circumstances. *The questions below were asked if the applicable precaution was not taken by the respondent.*

- You indicated that working from home was not one of the changes you made. Which of the following best describes why? [Choose one: I am retired/a student/currently not working; I could perhaps work from home, but it's better not to/I don't like to; I was not given the option to work from home (essential worker or employer needed me); I cannot work from home; Other: (fill in).]
- You indicated that wearing gloves when you go shopping was not one of the changes you made. Which of the following best describes why? [Choose one: It's not necessary; I would like to, but cannot find gloves; I don't shop for anything, including groceries; Other: (fill in).]

- You indicated that wearing masks was not one of the changes you made. Which of the following best describes why? [Choose one: Masks are not useful; I do not like wearing masks; I would like to, but cannot find or afford masks; I do not leave my house even to take a walk; Other: (fill in).]

- You indicated that avoiding all public places and self-isolating was not one of the changes you made. Which of the following best describes why? [Choose one: It's too much. We need to keep functioning; I would like to, but I have to work outside the home; I would like to, but I have to leave my house regularly for doctor visits; I would like to, but I have to go get food and groceries — I cannot afford to have it delivered; I would like to, but I have to go get food and groceries — I don't want to have it delivered; I would like to, but I have to go get food and groceries — there are no deliveries available; Other: (fill in).]

- You indicated that canceling travel plans was not one of the changes you made. Which of the following best describes why? [Choose one: I did not have any travel plans to begin with; I really wanted to go on the trip/did not feel like canceling; I could not get a refund and did not want to waste the money; I had to travel because of work obligations; I traveled because of family obligations; other: (fill in).]

- You indicated that avoiding large gatherings was not one of the changes you made. Which of the following best describes why? [Choose one: It's too much. We need to keep functioning; I would like to, but I cannot avoid large gatherings because of my work; Other: (fill in).]

Impact of the pandemic on the respondent, general

- Please rate how much trouble you are currently having with the following issues: [Issues: Having enough money for food and housing (rent, mortgage payments); Having enough money for medications; Having enough money for other expenses and/or savings; Finding groceries, even if you have the money to buy them; Finding medications, even if you have the money to buy them; Maintaining a positive outlook and not getting anxious; Mental health overall.] [Choose one: No trouble at all; Small problems; Some difficulties; Many difficulties; Serious trouble.]

News sources

- Which news sources do you usually rely on? [Choose all that apply: ABC News; CNN; Fox News Channel; Local news; NBC / MSNBC; NPR (Public Radio); Huff Post; The New York Times; The Wall Street Journal; Washington Post; Other: (fill in); I don't follow any news.]

- What other sources of information do you mostly pay attention to regarding the pandemic? [My friends; My family members; My pastor and/or our spiritual community; The President and his administration; Our Governor; Scientists/researchers; CDC (Center for Disease Control); People I follow on Twitter; People I follow on Facebook.] [Choose one: Not at all; A little bit; Somewhat; Mostly; Very much so; Not applicable.]

The study ended with inviting comments, if respondents had any.
